# Sterblichkeit bei Sepsis und septischem Schock in Deutschland. Ergebnisse eines systematischen Reviews mit Metaanalyse

**DOI:** 10.1007/s00101-021-00917-8

**Published:** 2021-02-09

**Authors:** Michael Bauer, Heinrich Volker Groesdonk, Franziska Preissing, Petra Dickmann, Tobias Vogelmann, Herwig Gerlach

**Affiliations:** 1 Klinik für Anästhesiologie und Intensivmedizin, Universitätsklinikum Jena, Am Klinikum 1, 07747 Jena, Deutschland; 2 Klinik für Interdisziplinäre Intensivmedizin und Intermediate Care, Helios Klinikum Erfurt, Nordhäuser Straße 74, 99089 Erfurt, Deutschland; 3 CytoSorbents Europe GmbH, Müggelseedamm 131, 12587 Berlin, Deutschland; 4 LinkCare GmbH, Kyffhäuserstr. 64, 70469 Stuttgart, Deutschland; 5 Klinik für Anästhesie, operative Intensivmedizin und Schmerztherapie, Vivantes Klinikum Neukölln, Rudower Straße 48, 12351 Berlin, Deutschland

**Keywords:** Mortalität, Sepsis, Infektion, Internationaler Vergleich, Metaanalyse, Mortality, Sepsis, Infection, International comparison, Systematic analysis

## Abstract

**Hintergrund:**

Verschiedene Autoren diskutieren, ob fehlende Qualitätsinitiativen und Behandlungsstandards in Deutschland im internationalen Vergleich zu höherer Sterblichkeit bei Sepsis und septischem Schock führen könnten. Dem gegenüber steht eine international anerkannte intensivmedizinische Versorgung in Deutschland, z. B. während der COVID-19-Pandemie.

**Ziel der Arbeit:**

Ziel dieser Studie war es, die Sterblichkeit bei Sepsis und septischem Schock in Deutschland zu ermitteln und mit anderen Industrienationen zu vergleichen.

**Material und Methoden:**

In eine systematischen Literaturrecherche wurden alle zwischen 2009 und 2020 veröffentlichten Interventions- und Beobachtungsstudien aus den Datenbanken PubMed und Cochrane Library eingeschlossen. Die 30- und 90-Tages-Sterblichkeit bei Sepsis und septischem Schock wurde in einer Metaanalyse mittels „Random-effects“-Modells gepoolt.

**Ergebnisse:**

Insgesamt wurden 134 Studien in die Meta-Analyse eingeschlossen. Die 30-Tages-Sterblichkeit bei Sepsis betrug in Deutschland 26,50 % (95 %-KI: 19,86–33,15 %), in Europa (ohne Deutschland) 23,85 % (95%-KI: 20,49–27,21 %) und in Nordamerika 19,58 % (95%-KI: 14,03–25,14 %). Die 30-Tages-Sterblichkeit bei septischem Schock betrug 30,48 % (95 %-KI: 29,30–31,67 %), 34,57 % (95 %-KI: 33,51–35,64 %) bzw. 33,69 % (95 %-KI: 31,51–35,86 %). Die 90-Tages-Sterblichkeit bei septischem Schock betrug 38,78 % (95 %-KI: 32,70–44,86 %), 41,90 % (95 %-KI: 38,88–44,91 %) beziehungsweise 34,41 % (95 %-KI: 25,66–43,16 %).

**Diskussion:**

Es ergaben sich somit keine Anhaltspunkte dafür, dass die Sterblichkeit bei Sepsis/septischem Schock im internationalen Vergleich in Deutschland erhöht ist.

**Zusatzmaterial online:**

Die Online-Version dieses Beitrags (10.1007/s00101-021-00917-8) enthält zusätzliche Informationen zu den eingeschlossenen Studien. Beitrag und Zusatzmaterial stehen Ihnen auf www.springermedizin.de zur Verfügung. Bitte geben Sie dort den Beitragstitel in die Suche ein, das Zusatzmaterial finden Sie beim Beitrag unter „Ergänzende Inhalte“.

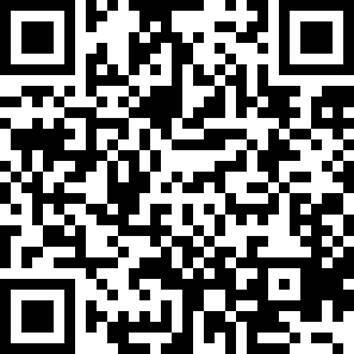

## Abstract (en)

**Background:**

The reported mortality for sepsis and septic shock varies between 15% and 59% in international comparison. For Germany, the number of studies is limited. Previous estimations of mortality in Germany are outdated or based on claims data analyses. Various authors discuss whether lacking quality initiatives and treatment standards in Germany could cause higher mortality for sepsis. This contrasts with the internationally well-recognized performance of the German intensive care infrastructure during the COVID-19 pandemic.

**Objectives:**

The objectives of this systematic review and meta-analysis were to estimate 30-day and 90-day mortality of patients with sepsis and patients with septic shock in Germany and to compare the mortality with that of other industrialized regions (Europe, North America).

**Material and methods:**

A systematic literature search included interventional and observational studies published between 2009 and 2020 in PubMed and the Cochrane Library that analyzed adult patients with sepsis, severe sepsis and septic shock in Europe and North America. Studies with less than 20 patients were excluded. The 30-day and 90-day mortality for sepsis and septic shock were pooled separately for studies conducted in Germany, Europe (excluding Germany) and North America in a meta-analysis using a random effects model. Mortality over time was analyzed in a linear regression model.

**Results:**

Overall, 134 studies were included. Of these, 15 studies were identified for the estimation of mortality in Germany, covering 10,434 patients, the number of patients per study ranged from 28 to 4183 patients. The 30-day mortality for sepsis was 26.50% (95% confidence interval, CI: 19.86–33.15%) in Germany, 23.85% (95% CI: 20.49–27.21%) in Europe (excluding Germany) and 19.58% (95% CI: 14.03–25.14%) in North America. The 30-day mortality for septic shock was 30.48% (95% CI: 29.30–31.67%) in Germany, 34.57% (95% CI: 33.51–35.64%) in Europe (excluding Germany) and 33.69% (95% CI: 31.51–35.86%) in North America. The 90-day mortality for septic shock was 38.78% (95% CI: 32.70–44.86%) in Germany, 41.90% (95% CI: 38.88–44.91%) in Europe (excluding Germany) and 34.41% (95% CI: 25.66–43.16%) in North America. A comparable decreasing trend in sepsis 30-day mortality was observed in all considered regions since 2009.

**Conclusion:**

Our analysis does not support the notion that mortality related to sepsis and septic shock in Germany is higher in international comparison. A higher mortality would not be obvious either, since intensive care, for example also during the COVID-19 pandemic, is regarded as exemplary in Germany and the structural quality, such as the number of intensive care beds per 100,000 inhabitants, is high in international comparison. Nevertheless, deficits could also exist outside intensive care medicine. A comparison of international individual studies should take greater account of the structure of healthcare systems, the severity of disease and the limitations resulting from the data sources used.

Laut Diagnosis Related Groups(DRG)-Abrechnungsdaten erleiden rund 300.000 Menschen/Jahr in Deutschland eine Sepsis [10, 12], wobei die Inzidenz um bis zu 15 % jährlich steigt [11]. Immer wieder wird diskutiert, ob die Sterblichkeit in Deutschland bei Sepsis höher liege als in anderen Industrienationen; die Schätzungen für diese Differenz liegen für die schwere Sepsis absolut bei 10–20 % [10, 12]. Für diese potenziell erhöhte Sterblichkeit werden fehlende Qualitätsinitiativen zur Früherkennung, die fehlende Verbesserung von Behandlungsstandards und fehlende Maßnahmen zur Reduktion von Krankenhausinfektionen, möglicherweise auch abseits der intensivmedizinischen Versorgung, verantwortlich gemacht [20].

## Hintergrund und Fragestellung

Bislang liegen keine systematischen Auswertungen vor, die über einen Vergleich von Einzelstudien hinaus die Sterblichkeit zwischen Deutschland und anderen Gesundheitssystemen quantifizieren. Die berichteten Sterblichkeitsangaben schwanken im internationalen Vergleich zwischen 15 und 59 % [1, 12, 13, 17, 23, 26]. Hierbei dürfte die unzureichende Berücksichtigung der Erkrankungsschwere (wie Einschluss von Patienten mit Sepsis und septischem Schock [3, 12]) zu den erheblichen Unterschieden in der Sterblichkeit zwischen den Studien – und somit auch zwischen verschiedenen Ländern – beitragen.

Wenig Beachtung findet in diesem Zusammenhang der Unterschied der Versorgungsstrukturen: Die Krankenhauskapazität, insbesondere die Kapazität der Intensivbetten, war in Deutschland schon vor der COVID-19-Pandemie fast doppelt so hoch wie der Durchschnitt der OECD-Länder [19]. Unstrittig geht mit dieser höheren Intensivkapazität ein unterschiedlicher Zugang zu Ressourcen der klinischen Versorgung, insbesondere für Patienten mit höherem Risiko (z. B. Hochbetagte), einher. Hier bestehen signifikante Unterschiede zwischen den OECD-Staaten, wobei in Deutschland eine „Deckelung“ des Zugangs aufgrund des Alters zur Intensivmedizin stärker als z. B. in Großbritannien abgelehnt wird [8, 15, 27]. Ein prominentes Beispiel ist die elektive Chirurgie, die in Großbritannien für Hochbetagte beschränkt [33], in Deutschland aber voll zugänglich ist und deren Sterblichkeit maßgeblich von septischen Komplikationen bestimmt wird [21]. Dies kann zur Überschätzung der Sterblichkeit, z. B. an nosokomialen Infektionen, in Deutschland im internationalen Vergleich beitragen.

Während die (intensiv-)medizinische Versorgung septischer Patienten in Deutschland im internationalen Vergleich als unterdurchschnittlich diskutiert wird, wurde trotz schwacher Datenlage das Management schwerer Verläufe von COVID-19, als aktuelles Beispiel eines septischen Krankheitsbildes [32], in Deutschland in der öffentlichen Diskussion als vorbildlich bezeichnet [5]. Auch bei solchen Vergleichen ist Vorsicht geboten, da viele Faktoren, z. B. das Alter der betroffenen Patientenkollektive, als wichtige Einflussgrößen variieren können [31]. Bei Beschränkung der Betrachtung auf die invasiv beatmeten Patienten sind ähnliche Sterblichkeiten für Deutschland und Großbritannien berichtet worden [16]. Die ausbleibende Überforderung der Gesundheitssysteme dürfte damit auch auf eine gute Verfügbarkeit und Strukturqualität in der Intensivmedizin zurückzuführen sein, die für den günstigen Verlauf der Pandemie in Deutschland zumindest mitverantwortlich ist. Die intensivmedizinische Strukturqualität wurde jedoch bisher als Einflussfaktor in vergleichenden Studien zur Sterblichkeit bestimmter Krankheitsbilder über Gesundheitssysteme hinweg zu wenig beachtet.

Defizite der Sepsisversorgung können auch abseits der Intensivmedizin bestehen. Eine diagnostische Unterversorgung in der Notaufnahme oder der Normalstation kann beispielsweise dazu führen, dass die Sepsis oder der septische Schock spät erkannt wird und Patienten dadurch mit erhöhter Krankheitsschwere auf die Intensivstation verlegt werden. Zusammengefasst fehlt bislang eine systematische Erfassung der Sterblichkeit der Sepsis bzw. des septischen Schocks in Deutschland, die eine Einordnung der Sterblichkeit im internationalen Vergleich und somit Hinweise auf Defizite in der (intensiv)medizinischen Versorgung ermöglicht.

Ziele dieses systematischen Reviews und dieser Metaanalyse waren daher zum einen die Schätzung der 30- und 90-Tages-Sterblichkeit von Patienten mit Sepsis sowie von Patienten mit septischem Schock in Deutschland und zum anderen die Gegenüberstellung der Sterblichkeit mit der vergleichbarer Regionen (Europa, Nordamerika).

## Studiendesign und Untersuchungsmethoden

Diese Studie basierte auf Daten der kürzlich publizierten Metaanalyse von Bauer et al. [3], in der wir die Sepsissterblichkeit in Europa und Nordamerika systematisch erhoben haben. Sterblichkeit ist in dieser Studie im Sinne von Letalität gemeint, die den Anteil der durch eine Krankheit Verstorbenen an der Zahl aller an dieser Krankheit Erkrankten angibt. Im Rahmen der vorliegenden Arbeit wurden diese Daten erweitert und separat nach Studien aus Deutschland stratifiziert. Die Datenbankrecherche wurde von Februar bis März 2019 auf PubMed und in der Cochrane Library durchgeführt. Studien wurden eingeschlossen, wenn (i) bei erwachsenen Patienten eine Sepsis, schwere Sepsis oder ein septischer Schock nach der Definition von Bone et al. [7] oder der Sepsis-3-Definition [28] vorlag, (ii) die 30(±2 Tage)- oder 90-Tages-Sterblichkeit berichtet wurde, (iii) diese zwischen 2009 und 2019 in (iv) englischer Sprache veröffentlicht und (v) in Europa oder Nordamerika durchgeführt wurden. Ausgeschlossen wurden Studien mit weniger als 20 Patienten und solche, die lediglich sozioökonomische Fragestellungen fokussierten. Eine ausführliche Beschreibung des Studiendesigns wurde bereits berichtet [3].

Ergänzend wurden für die vorliegende Analyse in Deutschland durchgeführte Studien, die zwischen April 2019 und Mai 2020 veröffentlicht wurden, gesucht.

Zur Auswertung der Endpunkte und der Analyse im Zeitverlauf wurden ein univariates „Random-effects“-Modell herangezogen und die Sterblichkeit im Zeitverlauf in einem linearen Regressionsmodell untersucht. Die Endpunkte wurden anhand der Region, in der die Studie durchgeführt wurde, analysiert, dabei wurde zwischen Deutschland, Europa (ohne Deutschland) und Nordamerika unterschieden. Mehrländerstudien, die keiner der genannten Regionen zugeordnet werden konnten, wurden nicht ausgewertet. Das Verzerrungsrisiko wurde für randomisierte Studien anhand des „The Cochrane Collaboration’s Tool RoB 2“ [30] und für Beobachtungsstudien anhand des „ROBINS-I“ erhoben [29].

## Ergebnisse

### Studieneinschluss

**Figure 1:**
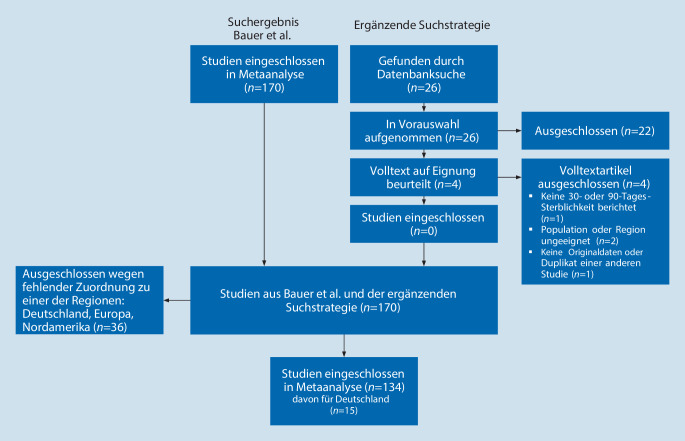

Die ursprüngliche Suchstrategie erzielte 4500 Treffer, von denen 170 Studien in die Metaanalyse eingeschlossen wurden. Die ergänzende Suchstrategie für deutsche Studien ergab keine neuen Studieneinschlüsse, weshalb die 170 Studien als Datengrundlage dienten. Davon wurden 36 Studien ausgeschlossen, da sie keiner der Zielregionen exklusiv zugeordnet werden konnten. Die Metaanalyse basierte auf den 134 verbliebenen Studien (Abb. 1). Für die Schätzung der Sterblichkeit in Deutschland wurden davon 15 Studien identifiziert.

### Studiencharakteristika

Die eingeschlossenen Studien zur Schätzung der Endpunkte in Deutschland umfassten insgesamt 10.434 Patienten. Die Patientenzahlen lagen zwischen 28 und 4183 Patienten/Studie. Das durchschnittliche Alter der Patienten lag bei 67,6 Jahren (Median: 65,6 Jahre); 8 der 15 Studien waren prospektive Kohortenstudien, 5 Studien waren RCT, und 2 Studien waren retrospektive Kohortenstudien (Zusatzmaterial online: Tabelle: Synopse der eingeschlossenen Studien).

### Verzerrungspotenzial

Die Bewertung des Verzerrungspotenzials je Studie ist für die 15 in Deutschland durchgeführten Studien einzeln dargestellt (Zusatzmaterial online, Zusammenstellung: „risk of bias assessment“ der eingeschlossenen Studien). Dabei hatten 7 Studien ein geringes, 7 Studien ein moderates und eine Studie ein hohes Verzerrungspotenzial.

### Metaanalyse

#### 30-Tages-Sterblichkeit bei Sepsis

**Figure 2:**
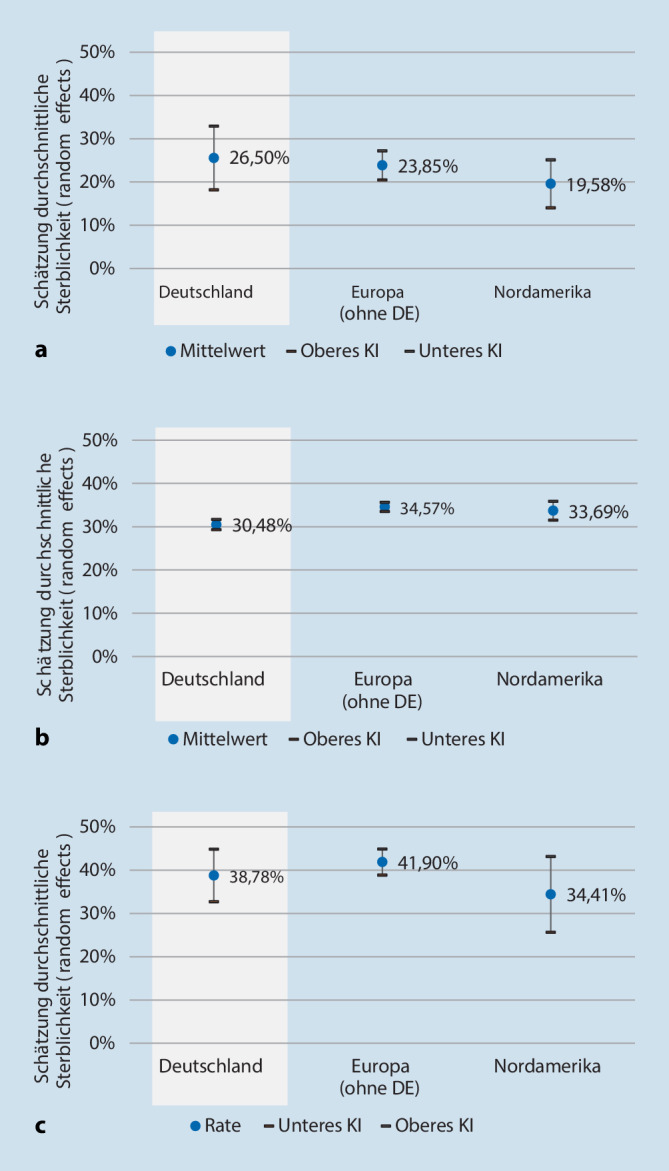

Die 30-Tages-Sterblichkeit bei Sepsis wurde für Deutschland auf 26,50 % (95 %-KI: 19,86–33,15 %) geschätzt. Das Heterogenitätsmaß I^2^ von 95,97 zeigte eine hohe Heterogenität an. Diese Analyse basierte auf 10 Studien mit insgesamt 7674 Patienten. Für Europa (ohne Deutschland) lag die Sterblichkeit mit 23,85 % (31 Studien; 16.904 Patienten; 95 %-KI: 20,49–27,21 %) etwa einen Prozentpunkt höher und für Nordamerika mit 19,58 % (16 Studien; 19.268 Patienten; 95 %-KI: 14,03–25,14 %) etwa 3 Prozentpunkte niedriger (Abb. 2).

Aufgrund der geringen Studienzahl konnte keine Analyse der 90-Tages-Sterblichkeit bei Sepsis durchgeführt werden.

#### 30-Tages-Sterblichkeit bei septischem Schock

Die 30-Tages-Sterblichkeit bei septischem Schock wurde für Deutschland auf 30,48 % (95 %-KI: 29,30–31,67 %) geschätzt. Das Heterogenitätsmaß I^2^ von 76,70 zeigte eine hohe Heterogenität an. Diese Analyse basierte auf 7 Studien mit insgesamt 8315 Patienten. Die Sterblichkeit lag damit leicht unter den Angaben für Europa (ohne Deutschland) mit 34,57 % (41 Studien; 11.784 Patienten; 95 %-KI: 33,51–35,64 %) und für Nordamerika mit 33,69 % (12 Studien; 2724 Patienten; 95 %-KI: 31,51–35,86 %), wobei sich die 95 %-Konfidenzintervalle nicht überschneiden.

#### 90-Tages-Sterblichkeit bei septischem Schock

Die 90-Tages-Sterblichkeit bei septischem Schock wurde für Deutschland auf 38,78 % (95 %-KI: 32,70–44,86 %) geschätzt. Das Heterogenitätsmaß I^2^ von 65,48 zeigte eine hohe Heterogenität an. Diese Analyse basierte auf 5 Studien mit insgesamt 3144 Patienten. Die Sterblichkeit für Europa (ohne Deutschland) lag mit 41,90 % (21 Studien; 14.936 Patienten; 95 %-KI: 38,88–44,91 %) etwa 3 Prozentpunkte höher, die Sterblichkeit in Nordamerika mit 34,41 % (3 Studien; 1493 Patienten; 95 %-KI: 25,66–43,16 %) etwa 4 Prozentpunkte darunter.

#### Entwicklung der Sterblichkeit im Zeitverlauf

**Figure 3:**
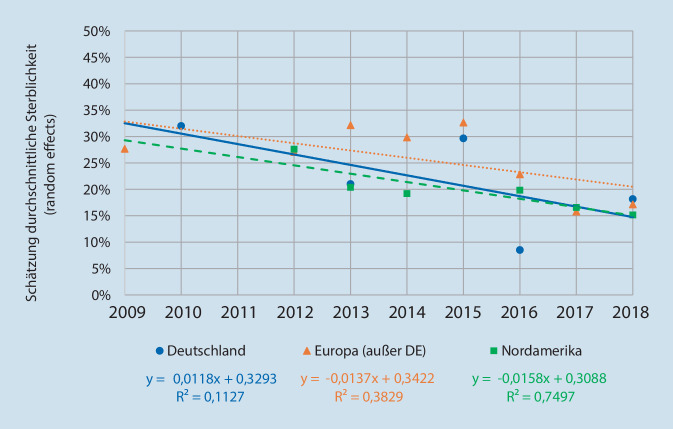

Die 30-Tages-Sterblichkeit bei Sepsis nahm im Zeitverlauf in allen betrachteten Regionen ab (Abb. 3). Tendenziell verläuft die Trendline für Deutschland etwas steiler fallend. Allerdings basierte diese Auswertung auf einer geringen Anzahl an Studien pro Jahr und Region, weshalb auf eine Prüfung der statistischen Signifikanz und der Darstellung weiterer Endpunkte im Zeitverlauf verzichtet wurde.

## Diskussion

Diese Metaanalyse ermittelte die durchschnittliche Sterblichkeit bei Sepsis bzw. septischem Schock in Deutschland nach 30 und 90 Tagen. Für die Sepsis wurde eine 30-Tages-Sterblichkeit von 26,50 % ermittelt.

Die hier geschätzte Sterblichkeit bei septischem Schock, insbesondere die 90-Tages-Sterblichkeit, ist mit den Ergebnissen der prospektiven SepNet-Beobachtungsstudie, die in 95 deutschen Krankenhäusern durchgeführt wurde und eine Krankenhaussterblichkeit bei schwerer Sepsis und septischem Schock von 40,4 % berichtete, vergleichbar [24]. Allerdings liegt die 30-Tages-Sterblichkeit darunter. Diese Abweichung könnte in den unterschiedlichen Studiendesigns begründet sein, da die Sterblichkeit in RCT, aufgrund engerer Einschlusskriterien, geringer als in anderen prospektiven oder retrospektiven Studiendesigns ist [3]. Die Schätzung der 30-Tages-Sterblichkeit basiert zu 70 % auf Patienten aus RCT. Im Gegensatz dazu berichteten die retrospektiven Arbeiten von Fleischmann et al. [12] und Engel et al. [9] eine deutlich höhere Sterblichkeit bei septischem Schock in Höhe von 58,8 % bzw. 55,2 %. Die Diskrepanz zu den Ergebnissen von Fleischmann et al. könnte mehrere Ursachen haben: Die von Fleischmann et al. verwendete DRG-Statistik diente primär der Abrechnung gegenüber den Krankenkassen, wodurch eine mögliche Verzerrung der Daten durch Vergütungsanreize (Überkodierung) nicht ausgeschlossen werden kann.

Verzerrungen durch Vergütungsanreize können nicht nur die berichtete Sterblichkeit bei septischem Schock beeinflussen, sondern auch zu einer Überschätzung der Patientenanzahl mit der milderen Form, der Sepsis, führen. Fleischmann et al. berichteten eine Krankenhaussterblichkeit von 24,3 % für Patienten mit Sepsis, inklusive schwerer Sepsis und septischem Schock [12]. Dieser Wert ist niedriger als erwartet. Grund hierfür könnte sein, dass Vergütungsanreize bestehen, die zur Kodierung leichter Sepsisverläufe, z. B. ohne Organfunktionsstörungen, motivieren. Dies würde zur Überschätzung der Sepsisinzidenz führen und hätte somit eine geringe Sterblichkeitsrate zur Folge.

Ähnliche Hinweise auf eine höhere Sterblichkeit in Abrechnungsdaten finden sich auch im Ausland: Rhee et al. verglichen die Sterblichkeit auf Basis von klinischen Patientenakten („electronic health records“) und Abrechnungsdaten (explizite ICD-Codes für schwere Sepsis und septischen Schock) von 173.690 Patienten in den USA. Die Sterblichkeit, basierend auf Abrechnungsdaten, lag je nach Jahr 10 bis 15 Prozentpunkte über der Schätzung, basierend auf klinischen Parametern aus Patientenakten. Andererseits schätzten Rhee et al. eine höhere Sepsisinzidenz, basierend auf klinischen Daten im Vergleich zu Abrechnungsdaten [23]. Es besteht daher weiterer Forschungsbedarf, die genauen Gründe dieser Abweichungen und den Einfluss von Kodieranreizen auf die Sepsisinzidenz gesondert je landesspezifischem Gesundheits-/und Vergütungssystem zu beurteilen.

Die Autoren kommen zu der Schlussfolgerung, dass patientenaktenbasierte klinische Daten für die Sepsis objektivere Schätzungen ermöglichen könnten als Abrechnungsdaten. Auch methodisch aufwendige Schätzungen der Sterblichkeit mittels Abrechnungsdaten, beispielsweise mittels „propensity score matching“ und Vergleichsgruppen, sind mit zahlreichen Limitationen verbunden [14].

Der Vergleich zu den Ergebnissen von Engel et al., deren Daten im Jahr 2003 erhoben wurden, könnte zum einen durch die Outcome-Erhebung begründet sein. Engel et al. erhoben die ICU- und Krankenhaussterblichkeit. Andere Studien zeigten, dass die ICU- und Krankenhaussterblichkeit 2 bis 6 Prozentpunkte über der 28-Tages-Sterblichkeit liegt, was einen Teil der Diskrepanz zu den hier geschätzten Ergebnissen erklären könnte [2]. Zum anderen kann der Unterschied ein Hinweis auf eine gesunkene Sterblichkeit bei septischem Schock sein. Allerdings müsste dieser Rückgang in den Jahren 2003 bis 2010 stattgefunden haben, da die Sterblichkeit bei septischem Schock im letzten Jahrzehnt nahezu konstant blieb [3].

Insgesamt konnte in diesem systematischen Review der letzten 10 Jahre lediglich eine Publikation gefunden werden, die eine ähnlich hohe 30-Tages-Sterblichkeit wie Fleischmann et al. und Engel et al. berichtete. Dies ist retrospektive Studie von Behnes et al. mit 57,0 %, die mit 74 Patienten eine kleine Stichprobe untersuchte [4].

Unsere Metaanalyse fand keine Hinweise, dass es Unterschiede in der Sterblichkeit zwischen Deutschland, Europa (ohne Deutschland) und Nordamerika gibt, die Konfidenzintervalle der einzelnen Endpunkte (30-/90-Tages-Sterblichkeit bei Sepsis bzw. septischem Schock) überschnitten sich mit Ausnahme der 30-Tages-Sterblickeit bei septischem Schock zwischen Deutschland und Europa ohne Deutschland. Keine Region hebt sich durch deutlich höhere bzw. niedrigere Sterblichkeitsangaben ab. Die Daten liefern daher keinen Anhaltspunkt zur Unterstützung der These, dass die Sterblichkeit in Deutschland im Vergleich zu anderen Regionen überdurchschnittlich sei. Weiteren Forschungsbedarf könnte ein Vergleich der Sepsisversorgung in Deutschland zu anderen Industrienationen je nach Setting (innerhalb und außerhalb der Intensivstation) darstellen.

Unsere Arbeit bestätigt somit die Aussagen vorheriger Metaanalysen zu ähnlichen Themen: Auch Vincent et al. berichteten in einer Metaanalyse keine statistisch signifikanten Unterschiede der Sterblichkeit bei septischem Schock in hochentwickelten Industrieländern (dort zwischen Europa und Nordamerika, 28-/30-Tages-Sterblichkeit in Europa: 38,5 %; in Nordamerika: 33,2 %) [34]. Levy et al. berichteten eine höhere Sterblichkeit in Europa als in den USA. Bei Adjustierung der Werte nach Erkrankungsschwere, die in Europa höher lag, ergab sich allerdings kein statistisch signifikanter Unterschied [18].

Nicht nur die Berücksichtigung der Erkrankungsschwere scheint im Studienvergleich insbesondere zwischen Europa und den USA erforderlich, auch die Berücksichtigung struktureller Unterschiede, wie der unterschiedlichen Verweildauern auf der Intensivstation, ist elementar. Die häufig berichtete Sterblichkeit auf der Intensivstation („ICU mortality“) [6] stellt zwar einen leicht zu erhebenden Endpunkt dar, ist allerdings international nur sehr eingeschränkt vergleichbar. Bei Ländern mit einer höheren Kapazität an Intensivbetten wird man tendenziell längere Verweildauern auf der Intensivstation erwarten, was mit einem höheren Sterblichkeitsrisiko aufgrund einer längeren Beobachtungszeit („detection bias“) einhergehen kann [22]. Da in Europa bei schwerer Sepsis und septischem Schock die Verweildauer mit 7,8 Tagen signifikant über der der USA von 4,2 Tagen liegt, ist beim Vergleich der ICU mortality Vorsicht geboten [18]. Diese Verzerrung kann durch den robusteren Vergleich der 30- bzw. 90-Tagessterblichkeit vermieden werden.

Diese Studie unterliegt einigen Limitationen. Die Studiensuche war auf die Datenbanken PubMed und Cochrane Library und auf englischsprachige Studien begrenzt. Die Einschlusskriterien waren weit gefasst, beispielsweise bezogen auf die Studiendesigns und Outcomes (28- und 30-Tages-Sterblichkeit), was mitunter zu hohen Heterogenitätswerten in der Metaanalyse führte.

Die im Jahr 2016 veröffentlichte neue Sepsisdefinition führte dazu, dass hier Studien, basierend auf der Sepsis-1/2-Definition und der Sepsis-3-Definition, eingeschlossen wurden [7, 25, 28]. Allerdings verwendete keine der deutschen Studien die Sepsis-3-Definition. Bezogen auf alle Studien lag der Anteil derer, die die Sepsis-3-Definition verwendeten, unter 5 %. Die Autoren schätzen die Verzerrung daher gering ein.

National wie international lassen sich zwar Hinweise auf im Zeitverlauf sinkende Sterblichkeitsraten ableiten, allerdings ist kein kontinuierlicher Rückgang der Sterblichkeit bei Sepsis und septischem Schock zu beobachten. Daraus könnte sich die Forderung nach Qualitätsinitiativen zur Früherkennung, Verbesserung von Behandlungsstandards und Maßnahmen zur Reduktion von Krankenhausinfektionen ergeben [3].

Zusammenfassend lässt sich für Deutschland im internationalen Vergleich keine höhere Sterblichkeit bei Sepsis bzw. septischem Schock feststellen. Eine höhere Sterblichkeit läge auch nicht nahe, da die Strukturqualität, wie die Zahl der Intensivbetten/100.000 Einwohner, im internationalen Vergleich hoch ist. Dennoch könnten auch abseits der Intensivmedizin Defizite bestehen. Ein Vergleich von internationalen Einzelstudien sollte neben dem Hintergrund der Erkrankungsschwere die Struktur der Gesundheitssysteme und die daraus resultierenden Limitationen der verwendeten Datenquellen stärker berücksichtigen.

## Fazit für die Praxis

- In Deutschland wurde die 30-Tages-Sterblichkeit bei Sepsis auf 26,50 % (95%-KI: 19,860–33,15 %) geschätzt.
- In Deutschland wurde die 30-Tages-Sterblichkeit bei septischem Schock auf 30,48 % (95 %-KI: 29,30–31,67 %) und die 90-Tages-Sterblichkeit auf 38,78 % (95 %-KI: 32,70–44,86 %) geschätzt.
- Diese Studie ergab keinen Anhaltpunkt dafür, dass sich die Sterblichkeit von Sepsis in Deutschland von anderen europäischen Ländern und Nordamerika unterscheidet.
- In allen betrachteten Regionen (Deutschland, restliches Europa und Nordamerika) ließ sich seit 2009 ein vergleichbarer, abnehmender Trend der Sepsissterblichkeit feststellen.
- Beim Vergleich internationaler Studienergebnisse und insbesondere bei der Analyse von Abrechnungsdaten bei Sepsis und septischem Schock sollten stärker die strukturellen Unterschiede in den Gesundheitssystemen und unterschiedlich lange Verweildauern auf Intensivstationen bzw. im Krankenhaus berücksichtigt werden.

## Supplementary Information




