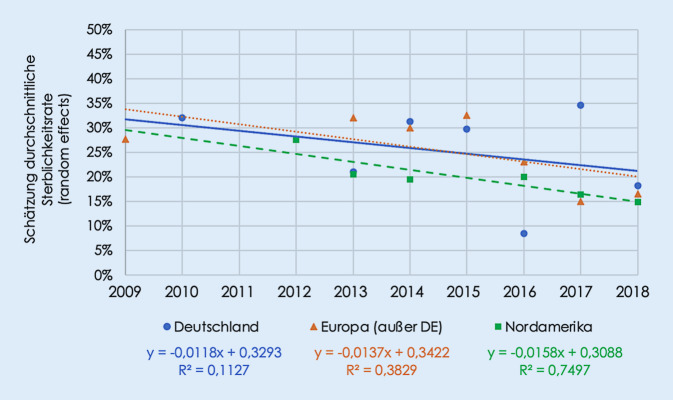# Erratum zu: Sterblichkeit bei Sepsis und septischem Schock in Deutschland. Ergebnisse eines systematischen Reviews mit Metaanalyse

**DOI:** 10.1007/s00101-021-00985-w

**Published:** 2021-06-21

**Authors:** Michael Bauer, Heinrich Volker Groesdonk, Franziska Preissing, Petra Dickmann, Tobias Vogelmann, Herwig Gerlach

**Affiliations:** 1 Klinik für Anästhesiologie und Intensivmedizin, Universitätsklinikum Jena, Am Klinikum 1, 07747 Jena, Deutschland; 2 Klinik für Interdisziplinäre Intensivmedizin und Intermediate Care, Helios Klinikum Erfurt, Erfurt, Deutschland; 3 CytoSorbents Europe GmbH, Berlin, Deutschland; 4 LinkCare GmbH, Stuttgart, Deutschland; 5 Klinik für Anästhesie, operative Intensivmedizin und Schmerztherapie, Vivantes Klinikum Neukölln, Berlin, Deutschland

**Erratum zu:**

**Anaesthesist 2021**

10.1007/s00101-021-00917-8

Die Autorinnen und Autoren weisen darauf hin, dass Daten zur 30-Tages-Sterblichkeit zu Sepsis in Deutschland nicht vollständig aus den genannten Studien extrahiert wurden.

Im *Abstract* muss es korrekterweise lauten „Insgesamt wurden 134 Studien in die Metaanalyse eingeschlossen. Die 30-Tages-Sterblichkeit bei Sepsis betrug in Deutschland 26,50 % (95 %-KI: 19,86–33,15 %), in Europa (ohne Deutschland) 23,85 % (95 %-KI: 20,49–27,21 %) und in Nordamerika 19,58 % (95 %-KI: 14,03–25,14 %)“.

Das *Ergebnis* wird wie folgt korrigiert „Die 30-Tages-Sterblichkeit bei Sepsis wurde für Deutschland auf 26,50 % (95 %-KI: 19,86–33,15 %) geschätzt. Das Heterogenitätsmaß I^2^ von 95,97 zeigte eine hohe Heterogenität an. Diese Analyse basierte auf 10 Studien mit insgesamt 7674 Patienten“.

Der dritte Satz der Diskussion muss richtigerweise heißen „Für die Sepsis wurde eine 30-Tages-Sterblichkeit von 26,50 % ermittelt.“

Im *Fazit für die Praxis* korrigiert sich der erste Punkt zu „In Deutschland wurde die 30-Tages-Sterblichkeit bei Sepsis auf 26,50 % (95 %-KI: 19,860–33,15 %) geschätzt.“

Abb. 2a und 3 sind korrigiert abgebildet.

**Figure 1:**
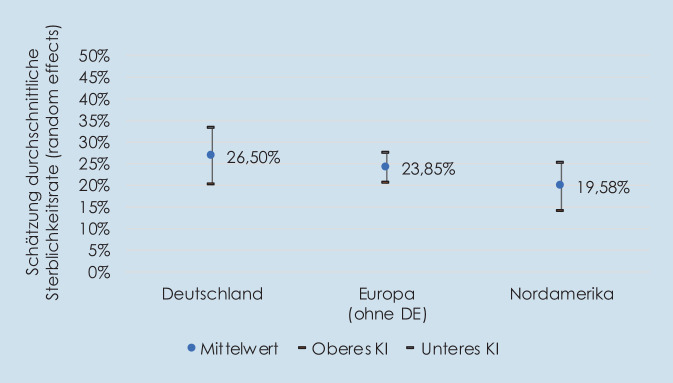

**Figure 2:**