# Supplementary material for: Mortality in sepsis and septic shock in Germany. Results of a systematic review and meta-analysis
Source: Anaesthesist. 2021 Feb 9;70(8):673–80. [Article in German] doi: 10.1007/s00101-021-00917-8 (PMC7871311; doi:10.1007/s00101-021-00917-8)
Supplement: Supplementary file 1 [file 101_2021_917_MOESM1_ESM.pdf]

Zusatzmaterial zum Beitrag „Sterblichkeit bei Sepsis und septischem Schock in Deutschland. Ergebnisse eines systematischen Reviews mit Meta-Analyse“ von Bauer M, Groesdonk HV, Preissing F et al. (2021) in *Der Anaesthesist.*
Beitrag und Zusatzmaterial stehen Ihnen auf www.springermedizin.de zur Verfügung. Bitte geben Sie dort den Beitragstitel in die Suche ein.

Tabelle - Synopse der eingeschlossenen Studien für Deutschland

	Autor, Jahr	Titel	Studientyp	Patienten-zahl	Betrachteter Endpunkt
1	Behnes, 2014	Diagnostic and prognostic utility of soluble CD 14 subtype (presepsin) for severe sepsis and septic shock during the first week of intensive care treatment.	Retrospektive Kohortenstudie	74	30-Tages-Sterblichkeit septischer Schock
2	Bloos, 2014	Impact of compliance with infection management guidelines on outcome in patients with severe sepsis: a prospective observational multi-center study.	Prospektive Kohortenstudie	1.011	30-Tages-Sterblichkeit septischer Schock
3	Bloos, 2016	Effect of Sodium Selenite Administration and Procalcitonin-Guided Therapy on Mortality in Patients With Severe Sepsis or Septic Shock: A Randomized Clinical Trial.	RCT	1.089	▪ 30-Tages-Sterblichkeit septischer Schock<br>▪ 90-Tages-Sterblichkeit septischer Schock
4	Bloos, 2017	Effect of a multifaceted educational intervention for anti-infectious measures on sepsis mortality: a cluster randomized trial.	RCT	4.183	30-Tages-Sterblichkeit septischer Schock
5	Brunkhorst, 2012	Effect of empirical treatment with moxifloxacin and meropenem vs	RCT	551	▪ 30-Tages-Sterblichkeit septischer Schock

		meropenem on sepsis-related organ dysfunction in patients with severe sepsis: a randomized trial.			▪ 90-Tages-Sterblichkeit septischer Schock
6	Elke, 2013	Enteral nutrition is associated with improved outcome in patients with severe sepsis. A secondary analysis of the VISEP trial.	Retrospektive Kohortenstudie	353	▪ 30-Tages-Sterblichkeit Sepsis<br>▪ 90-Tages-Sterblichkeit Sepsis
7	Kaffarnik, 2013	Early diagnosis of sepsis-related hepatic dysfunction and its prognostic impact on survival: a prospective study with the LiMAx test.	Prospektive Kohortenstudie	28	90-Tages-Sterblichkeit Sepsis
8	Keh, 2016	Effect of Hydrocortisone on Development of Shock Among Patients With Severe Sepsis: The HYPRESS Randomized Clinical Trial.	RCT	353	▪ 30-Tages-Sterblichkeit Sepsis<br>▪ 90-Tages-Sterblichkeit Sepsis
9	Koch, 2010	Regulation and prognostic relevance of serum ghrelin concentrations in critical illness and sepsis.	Prospektive Kohortenstudie	122	▪ 30-Tages-Sterblichkeit Sepsis<br>▪ 90-Tages-Sterblichkeit Sepsis
10	Kristof, 2018	Anaemia requiring red blood cell transfusion is associated with unfavourable 90-day survival in surgical patients with sepsis.	Prospektive Kohortenstudie	435	▪ 30-Tages-Sterblichkeit Sepsis<br>▪ 90-Tages-Sterblichkeit Sepsis
11	Mansur, 2015	Impact of statin therapy on mortality in patients with sepsis-associated acute respiratory distress syndrome (ARDS) depends on ARDS severity: a prospective observational cohort study.	Prospektive Kohortenstudie	404	▪ 30-Tages-Sterblichkeit Sepsis
12	Mansur, 2015	Primary bacteraemia is associated with a higher mortality risk compared with pulmonary and	Prospektive Kohortenstudie	327	▪ 30-Tages-Sterblichkeit Sepsis

		intra-abdominal infections in patients with sepsis: a prospective observational cohort study.			▪ 90-Tages-Sterblichkeit Sepsis
13	Schädler, 2017	The effect of a novel extracorporeal cytokine hemoadsorption device on IL-6 elimination in septic patients: A randomized controlled trial.	RCT	97	▪ 90-Tages-Sterblichkeit septischer Schock
14	Scheer, 2017	Quality Improvement Initiative for Severe Sepsis and Septic Shock Reduces 90-Day Mortality: A 7.5-Year Observational Study.	Prospektive Kohortenstudie	1.373	▪ 30-Tages-Sterblichkeit septischer Schock<br>▪ 90-Tages-Sterblichkeit septischer Schock
15	Simon, 2017	Plasma adrenomedullin in critically ill patients with sepsis after major surgery: A pilot study.	Prospektive Kohortenstudie	34	▪ 30-Tages-Sterblichkeit septischer Schock<br>▪ 90-Tages-Sterblichkeit septischer Schock
