# Supplementary material for: Mortality in sepsis and septic shock in Germany. Results of a systematic review and meta-analysis
Source: Anaesthesist. 2021 Feb 9;70(8):673–80. [Article in German] doi: 10.1007/s00101-021-00917-8 (PMC7871311; doi:10.1007/s00101-021-00917-8)
Supplement: Supplementary file 2 [file 101_2021_917_MOESM2_ESM.pdf]

Zusatzmaterial zum Beitrag „Sterblichkeit bei Sepsis und septischem Schock in Deutschland. Ergebnisse eines systematischen Reviews mit Meta-Analyse“ von Bauer M, Groesdonk HV, Preissing F et al. (2021) in *Der Anaesthesist.*
Beitrag und Zusatzmaterial stehen Ihnen auf www.springermedizin.de zur Verfügung. Bitte geben Sie dort den Beitragstitel in die Suche ein.

Zusammenstellung: Risk of Bias Assessment der eingeschlossenen Studien für Deutschland

Inhalt

Behnes, 2014 2
Bloos, 2014 10
Bloos, 2016 18
Bloos, 2017 29
Brunkhorst, 2012 39
Elke, 2013 48
Kaffarnik, 2013 56
Keh, 2016 66
Koch, 2010 75
Kristof, 2018 85
Mansur, 2015a 95
Mansur, 2015b 105
Schädler, 2017 115
Scheer, 2017 124
Simon, 2017 134

Behnes, 2014

Responses underlined in green are potential markers for low risk of bias, and responses in red are potential markers for a risk of bias. Where questions relate only to sign posts to other questions, no formatting is used.

Signalling questions	Description	Response options
**Bias due to confounding**		
1.1 Is there potential for confounding of the effect of intervention in this study?<br>**If N/PN to 1.1:** the study can be considered to be at low risk of bias due to confounding and no further signalling questions need be considered	PY	Y / PY / PN / N
**If Y/PY to 1.1**: determine whether there is a need to assess time-varying confounding:		
1.2. Was the analysis based on splitting participants' follow up time according to intervention received?<br>**If N/PN**, answer questions relating to baseline confounding (1.4 to 1.6)<br>**If Y/PY**, go to question 1.3.	N	NA / Y / PY / PN / N / NI
1.3. Were intervention discontinuations or switches likely to be related to factors that are prognostic for the outcome?<br>**If N/PN**, answer questions relating to baseline confounding (1.4 to 1.6)<br>**If Y/PY**, answer questions relating to both baseline and time-varying confounding (1.7 and 1.8)		NA / Y / PY / PN / N / NI

**Questions relating to baseline confounding only**		
1.4. Did the authors use an appropriate analysis method that controlled for all the important confounding domains?	PY	NA / Y / PY / PN / N / NI
1.5. **If Y/PY to 1.4**: Were confounding domains that were controlled for measured validly and reliably by the variables available in this study?	Y	NA / Y / PY / PN / N / NI
1.6. Did the authors control for any post-intervention variables that could have been affected by the intervention?	Y	NA / Y / PY / PN / N / NI
**Questions relating to baseline and time-varying confounding**		
1.7. Did the authors use an appropriate analysis method that controlled for all the important confounding domains and for time-varying confounding?	PY	NA / Y / PY / PN / N / NI
1.8. **If Y/PY to 1.7**: Were confounding domains that were controlled for measured validly and reliably by the variables available in this study?	Y	NA / Y / PY / PN / N / NI
**Risk of bias judgement**	Low to Moderate	Low / Moderate / Serious / Critical / NI
Optional: What is the predicted direction of bias due to confounding?		Favours experimental / Favours comparator / Unpredictable

Bias in selection of participants into the study		
2.1. Was selection of participants into the study (or into the analysis) based on participant characteristics observed after the start of intervention?<br>**If N/PN to 2.1:** go to 2.4	Y	Y / PY / PN / N / NI
2.2. **If Y/PY to 2.1**: Were the post-intervention variables that influenced selection likely to be associated with intervention?	N	NA / Y / PY / PN / N / NI
2.3 **If Y/PY to 2.2**: Were the post-intervention variables that influenced selection likely to be influenced by the outcome or a cause of the outcome?	N	NA / Y / PY / PN / N / NI
2.4. Do start of follow-up and start of intervention coincide for most participants?	PY	Y / PY / PN / N / NI
2.5. **If Y/PY to 2.2 and 2.3, or N/PN to 2.4**: Were adjustment techniques used that are likely to correct for the presence of selection biases?		NA / Y / PY / PN / N / NI
**Risk of bias judgement**	Moderate	Low / Moderate / Serious / Critical / NI
Optional: What is the predicted direction of bias due to selection of participants into the study?		Favours experimental / Favours comparator / Towards null /Away from null / Unpredictable

**Bias in classification of interventions**		
3.1 Were intervention groups clearly defined?	Y	Y / PY / PN / N / NI
3.2 Was the information used to define intervention groups recorded at the start of the intervention?	Y	Y / PY / PN / N / NI
3.3 Could classification of intervention status have been affected by knowledge of the outcome or risk of the outcome?	N	Y / PY / PN / N / NI
**Risk of bias judgement**	low	Low / Moderate / Serious / Critical / NI
Optional: What is the predicted direction of bias due to classification of interventions?		Favours experimental / Favours comparator / Towards null /Away from null / Unpredictable

Bias due to missing data		
5.1 Were outcome data available for all, or nearly all, participants?	PY	Y / PY / PN / N / NI
5.2 Were participants excluded due to missing data on intervention status?	PN	Y / PY / PN / N / NI
5.3 Were participants excluded due to missing data on other variables needed for the analysis?	NI	Y / PY / PN / N / NI
5.4 **If PN/N to 5.1, or Y/PY to 5.2 or 5.3**: Are the proportion of participants and reasons for missing data similar across interventions?	NI	NA / Y / PY / PN / N / NI
5.5 **If PN/N to 5.1, or Y/PY to 5.2 or 5.3**: Is there evidence that results were robust to the presence of missing data?	NI	NA / Y / PY / PN / N / NI
**Risk of bias judgement**	Low	Low / Moderate / Serious / Critical / NI
Optional: What is the predicted direction of bias due to missing data?		Favours experimental / Favours comparator / Towards null /Away from null / Unpredictable

Bias in measurement of outcomes		
6.1 Could the outcome measure have been influenced by knowledge of the intervention received?	N	Y / PY / PN / N / NI
6.2 Were outcome assessors aware of the intervention received by study participants?	NI	Y / PY / PN / N / NI
6.3 Were the methods of outcome assessment comparable across intervention groups?	Y	Y / PY / PN / N / NI
6.4 Were any systematic errors in measurement of the outcome related to intervention received?	PN	Y / PY / PN / N / NI
**Risk of bias judgement**	low	Low / Moderate / Serious / Critical / NI
Optional: What is the predicted direction of bias due to measurement of outcomes?		Favours experimental / Favours comparator / Towards null /Away from null / Unpredictable

Bias in selection of the reported result		
Is the reported effect estimate likely to be selected, on the basis of the results, from... 7.1. ... multiple outcome *measurements* within the outcome domain?		Y / PY / PN / N / NI
7.2 ... multiple *analyses* of the intervention-outcome relationship?	N	Y / PY / PN / N / NI
7.3 ... different *subgroups*?	N	Y / PY / PN / N / NI
**Risk of bias judgement**	Low to moderate	Low / Moderate / Serious / Critical / NI
Optional: What is the predicted direction of bias due to selection of the reported result?		Favours experimental / Favours comparator / Towards null /Away from null / Unpredictable

Overall bias		
**Risk of bias judgement**	Low to moderate	Low / Moderate / Serious / Critical / NI
Optional: What is the overall predicted direction of bias for this outcome?		Favours experimental / Favours comparator / Towards null /Away from null / Unpredictable

Bloos, 2014

Signalling questions	Description	Response options
**Bias due to confounding**		
1.1 Is there potential for confounding of the effect of intervention in this study?<br>**If N/PN to 1.1:** the study can be considered to be at low risk of bias due to confounding and no further signalling questions need be considered	PN	Y / PY / PN / N
**If Y/PY to 1.1**: determine whether there is a need to assess time-varying confounding:		
1.2. Was the analysis based on splitting participants' follow up time according to intervention received?<br>**If N/PN**, answer questions relating to baseline confounding (1.4 to 1.6)<br>**If Y/PY**, go to question 1.3.		NA / Y / PY / PN / N / NI
1.3. Were intervention discontinuations or switches likely to be related to factors that are prognostic for the outcome?<br>**If N/PN**, answer questions relating to baseline confounding (1.4 to 1.6)<br>**If Y/PY**, answer questions relating to both baseline and time-varying confounding (1.7 and 1.8)		NA / Y / PY / PN / N / NI

**Questions relating to baseline confounding only**		
1.4. Did the authors use an appropriate analysis method that controlled for all the important confounding domains?		NA / Y / PY / PN / N / NI
1.5. **If Y/PY to 1.4**: Were confounding domains that were controlled for measured validly and reliably by the variables available in this study?		NA / Y / PY / PN / N / NI
1.6. Did the authors control for any post-intervention variables that could have been affected by the intervention?		NA / Y / PY / PN / N / NI
**Questions relating to baseline and time-varying confounding**		
1.7. Did the authors use an appropriate analysis method that controlled for all the important confounding domains and for time-varying confounding?		NA / Y / PY / PN / N / NI
1.8. **If Y/PY to 1.7**: Were confounding domains that were controlled for measured validly and reliably by the variables available in this study?		NA / Y / PY / PN / N / NI
**Risk of bias judgement**		Low / Moderate / Serious / Critical / NI
Optional: What is the predicted direction of bias due to confounding?		Favours experimental / Favours comparator / Unpredictable

Bias in selection of participants into the study		
2.1. Was selection of participants into the study (or into the analysis) based on participant characteristics observed after the start of intervention?<br>**If N/PN to 2.1:** go to 2.4<br>2.2. **If Y/PY to 2.1**: Were the post-intervention variables that influenced selection likely to be associated with intervention?<br>2.3 **If Y/PY to 2.2**: Were the post-intervention variables that influenced selection likely to be influenced by the outcome or a cause of the outcome?	PN	Y / PY / PN / N / NI<br>NA / Y / PY / PN / N / NI<br>NA / Y / PY / PN / N / NI
2.4. Do start of follow-up and start of intervention coincide for most participants?	Y	Y / PY / PN / N / NI
2.5. **If Y/PY to 2.2 and 2.3, or N/PN to 2.4**: Were adjustment techniques used that are likely to correct for the presence of selection biases?		NA / Y / PY / PN / N / NI
**Risk of bias judgement**	Low	Low / Moderate / Serious / Critical / NI
Optional: What is the predicted direction of bias due to selection of participants into the study?		Favours experimental / Favours comparator / Towards null /Away from null / Unpredictable

Bias in classification of interventions		
3.1 Were intervention groups clearly defined?	Y	Y / PY / PN / N / NI
3.2 Was the information used to define intervention groups recorded at the start of the intervention?	Y	Y / PY / PN / N / NI
3.3 Could classification of intervention status have been affected by knowledge of the outcome or risk of the outcome?	PN	Y / PY / PN / N / NI
**Risk of bias judgement**	low	Low / Moderate / Serious / Critical / NI
Optional: What is the predicted direction of bias due to classification of interventions?		Favours experimental / Favours comparator / Towards null /Away from null / Unpredictable

Bias due to missing data		
5.1 Were outcome data available for all, or nearly all, participants?	PY	Y / PY / PN / N / NI
5.2 Were participants excluded due to missing data on intervention status?	Y	Y / PY / PN / N / NI
5.3 Were participants excluded due to missing data on other variables needed for the analysis?	Y	Y / PY / PN / N / NI
5.4 **If PN/N to 5.1, or Y/PY to 5.2 or 5.3**: Are the proportion of participants and reasons for missing data similar across interventions?	PY	NA / Y / PY / PN / N / NI
5.5 **If PN/N to 5.1, or Y/PY to 5.2 or 5.3**: Is there evidence that results were robust to the presence of missing data?	PY	NA / Y / PY / PN / N / NI
**Risk of bias judgement**	Moderate	Low / Moderate / Serious / Critical / NI
Optional: What is the predicted direction of bias due to missing data?		Favours experimental / Favours comparator / Towards null /Away from null / Unpredictable

Bias in measurement of outcomes		
6.1 Could the outcome measure have been influenced by knowledge of the intervention received?	N	Y / PY / PN / N / NI
6.2 Were outcome assessors aware of the intervention received by study participants?	NI	Y / PY / PN / N / NI
6.3 Were the methods of outcome assessment comparable across intervention groups?	Y	Y / PY / PN / N / NI
6.4 Were any systematic errors in measurement of the outcome related to intervention received?	PN	Y / PY / PN / N / NI
**Risk of bias judgement**	Low	Low / Moderate / Serious / Critical / NI
Optional: What is the predicted direction of bias due to measurement of outcomes?		Favours experimental / Favours comparator / Towards null /Away from null / Unpredictable

Bias in selection of the reported result		
Is the reported effect estimate likely to be selected, on the basis of the results, from...		
7.1. ... multiple outcome *measurements* within the outcome domain?	NI	Y / PY / PN / N / NI
7.2 ... multiple *analyses* of the intervention-outcome relationship?	NI	Y / PY / PN / N / NI
7.3 ... different *subgroups*?	NI	Y / PY / PN / N / NI
**Risk of bias judgement**	low	Low / Moderate / Serious / Critical / NI
Optional: What is the predicted direction of bias due to selection of the reported result?		Favours experimental / Favours comparator / Towards null /Away from null / Unpredictable

Overall bias		
**Risk of bias judgement**	low	Low / Moderate / Serious / Critical / NI
Optional: What is the overall predicted direction of bias for this outcome?		Favours experimental / Favours comparator / Towards null /Away from null / Unpredictable

Bloos, 2016

Study details

**Reference**	Bloos F et al, Effect of Sodium Selenite Administration and Procalcitonin-Guided Therapy on Mortality in Patients With Severe Sepsis or Septic Shock: A Randomized Clinical Trial. JAMA Intern Med. 2016 Sep 1;176(9):1266-76.

Study design

X Individually-randomized parallel-group trial
☐ Cluster-randomized parallel-group trial
☐ Individually randomized cross-over (or other matched) trial

For the purposes of this assessment, the interventions being compared are defined as

Experimental:	high-dose intravenous sodium selenite treatment (GROUP 1)	Comparator:	Placebo
Experimental:	procalcitonin-guided anti-infectious therapy (GROUP 2)	Comparator:	Standard anti-infectious therapy algorithm

**Specify which outcome is being assessed for risk of bias**	28-day mortality, %
**Specify the numerical result being assessed.** In case of multiple alternative analyses being presented, specify the numeric result (e.g. RR = 1.52 (95% CI 0.83 to 2.77) and/or a reference (e.g. to a	28.3% GROUP 1 vs. 25.5% placebo<br><br>25.6% GROUP 2 vs 28.2% no PCT guidance

table, figure or paragraph) that uniquely defines the result being assessed.

Is the review team's aim for this result…?

X to assess the effect of *assignment to intervention* (the 'intention-to-treat' effect)

☐ to assess the effect of *adhering to intervention* (the 'per-protocol' effect)

If the aim is to assess the effect of *adhering to intervention*, select the deviations from intended intervention that should be addressed (at least one must be checked):

☐ occurrence of non-protocol interventions

☐ failures in implementing the intervention that could have affected the outcome

☐ non-adherence to their assigned intervention by trial participants

Which of the following sources were <u>obtained</u> to help inform the risk-of-bias assessment? (tick as many as apply)

X Journal article(s) with results of the trial

X Trial protocol

X Statistical analysis plan (SAP)

X Non-commercial trial registry record (e.g. ClinicalTrials.gov record)

☐ Company-owned trial registry record (e.g. GSK Clinical Study Register record)

☐ "Grey literature" (e.g. unpublished thesis)

☐ Conference abstract(s) about the trial

☐ Regulatory document (e.g. Clinical Study Report, Drug Approval Package)

☐ Research ethics application

☐ Grant database summary (e.g. NIH RePORTER or Research Councils UK Gateway to Research)

☐ Personal communication with trialist

☐ Personal communication with the sponsor

Risk of bias assessment

Responses underlined in green are potential markers for low risk of bias, and responses in red are potential markers for a risk of bias. Where questions relate only to sign posts to other questions, no formatting is used.

Domain 1: Risk of bias arising from the randomization process

Signalling questions	Comments	Response options
**1.1 Was the allocation sequence random?**	Y	Y / PY / PN / N / NI
**1.2 Was the allocation sequence concealed until participants were enrolled and assigned to interventions?**	PY	Y / PY / PN / N / NI
**1.3 Did baseline differences between intervention groups suggest a problem with the randomization process?**	PN	Y / PY / PN / N / NI
**Risk-of-bias judgement**	Low	Low / High / Some concerns
Optional: What is the predicted direction of bias arising from the randomization process?		NA / Favours experimental / Favours comparator / Towards null /Away from null / Unpredictable

Domain 2: Risk of bias due to deviations from the intended interventions (*effect of assignment to intervention*)

Signalling questions	Comments	Response options
**2.1. Were participants aware of their assigned intervention during the trial?**	N (GROUP 1)<br>Y (GROUP 2)	Y / PY / PN / N / NI
**2.2. Were carers and people delivering the interventions aware of participants' assigned intervention during the trial?**	N (GROUP 1)<br>Y (GROUP 2)	Y / PY / PN / N / NI
**2.3. If Y/PY/NI to 2.1 or 2.2: Were there deviations from the intended intervention that arose because of the trial context?**	PN (GROUP 2)	NA / Y / PY / PN / N / NI
**2.4 If Y/PY to 2.3: Were these deviations likely to have affected the outcome?**		NA / Y / PY / PN / N / NI
**2.5. If Y/PY/NI to 2.4: Were these deviations from intended intervention balanced between groups?**		NA / Y / PY / PN / N / NI
**2.6 Was an appropriate analysis used to estimate the effect of assignment to intervention?**	Y	Y / PY / PN / N / NI
**2.7 If N/PN/NI to 2.6: Was there potential for a substantial impact (on the result) of the failure to analyse participants in the group to which they were randomized?**		NA / Y / PY / PN / N / NI
**Risk-of-bias judgement**	Low (both intervention groups)	Low / High / Some concerns

Optional: What is the predicted direction of bias due to deviations from intended interventions?		NA / Favours experimental / Favours comparator / Towards null /Away from null / Unpredictable

Domain 3: Missing outcome data

Signalling questions	Comments	Response options
**3.1 Were data for this outcome available for all, or nearly all, participants randomized?**	Y	Y / PY / PN / N / NI
**3.2 If N/PN/NI to 3.1: Is there evidence that the result was not biased by missing outcome data?**		NA / Y / PY / PN / N
**3.3 If N/PN to 3.2: Could missingness in the outcome depend on its true value?**		NA / Y / PY / PN / N / NI
**3.4 If Y/PY/NI to 3.3: Is it likely that missingness in the outcome depended on its true value?**		NA / Y / PY / PN / N / NI
**Risk-of-bias judgement**	Low	Low / High / Some concerns
Optional: What is the predicted direction of bias due to missing outcome data?		NA / Favours experimental / Favours comparator / Towards null /Away from null / Unpredictable

Domain 4: Risk of bias in measurement of the outcome

Signalling questions	Comments	Response options
**4.1 Was the method of measuring the outcome inappropriate?**	PN<br>We found a statistically significant interaction between the 2 study interventions regarding the primary end point (P = .03). Because there was no indication that selenium influenced the plasma procalcitonin levels or that procalcitonin guidance influenced the plasma selenium levels we decided to accept the observed statistical interaction as a chance finding and proceed with the factorial analysis as originally planned. However, we also report data in consideration of the significant interaction.	Y / PY / PN / N / NI
**4.2 Could measurement or ascertainment of the outcome have differed between intervention groups?**	PN	Y / PY / PN / N / NI
**4.3 If N/PN/NI to 4.1 and 4.2: Were outcome assessors aware of the intervention received by study participants?**	PN (Group 1)<br>PY (Group 2)	NA / Y / PY / PN / N / NI
**4.4 If Y/PY/NI to 4.3: Could assessment of the outcome have been influenced by knowledge of intervention received?**	PN	NA / Y / PY / PN / N / NI
**4.5 If Y/PY/NI to 4.4: Is it likely that assessment of the outcome was influenced by knowledge of intervention received?**		NA / Y / PY / PN / N / NI
**Risk-of-bias judgement**	Low	Low / High / Some concerns

Optional: What is the predicted direction of bias in measurement of the outcome?		NA / Favours experimental / Favours comparator / Towards null /Away from null / Unpredictable

Domain 5: Risk of bias in selection of the reported result

Signalling questions	Comments	Response options
**5.1 Were the data that produced this result analysed in accordance with a pre-specified analysis plan that was finalized before unblinded outcome data were available for analysis?**	Y	Y / PY / PN / N / NI
**Is the numerical result being assessed likely to have been selected, on the basis of the results, from...**		
**5.2. ... multiple eligible outcome measurements (e.g. scales, definitions, time points) within the outcome domain?**	N	Y / PY / PN / N / NI
**5.3 ... multiple eligible analyses of the data?**	PN	Y / PY / PN / N / NI
**Risk-of-bias judgement**	Low	Low / High / Some concerns
Optional: What is the predicted direction of bias due to selection of the reported result?		NA / Favours experimental / Favours comparator / Towards null /Away from null / Unpredictable

Overall risk of bias

**Risk-of-bias judgement**	Low	Low / High / Some concerns
Optional: What is the overall predicted direction of bias for this outcome?		NA / Favours experimental / Favours comparator / Towards null /Away from null / Unpredictable
Comments	Authors found a statistically significant interaction between the 2 study interventions regarding the primary end point (P = .03). Because there was no indication that selenium influenced the plasma procalcitonin levels or that procalcitonin guidance influenced the plasma selenium levels authors decided to accept the observed statistical interaction as a chance finding and proceed with the factorial analysis as originally planned. See ETable4.	

Bloos, 2017

**Study details**	
**Reference**	Bloos F et al, Effect of a Multifaceted Educational Intervention for Anti-Infectious Measures on Sepsis Mortality: A Cluster Randomized Trial. Intensive Care Med. 2017 Nov;43(11):1602-1612.

Study design

- ☐ Individually-randomized parallel-group trial
- X Cluster-randomized parallel-group trial
- ☐ Individually randomized cross-over (or other matched) trial

For the purposes of this assessment, the interventions being compared are defined as

Experimental:	multifaceted interventions including local quality improvement teams, educational outreach, audit, feedback, and reminders	Comparator:	conventional continuous medical education (CME) measures

**Specify which outcome is being assessed for risk of bias**	28-day mortality, %
**Specify the numerical result being assessed.** In case of multiple alternative analyses being presented, specify the numeric result (e.g. RR = 1.52 (95% CI 0.83 to 2.77) and/or a reference (e.g. to a	35.1% (883 of 2596 patients) vs. 26.7% (403 of 1587 patients; p = 0.01)

table, figure or paragraph) that uniquely defines the result being assessed.

Is the review team's aim for this result…?

X to assess the effect of *assignment to intervention* (the 'intention-to-treat' effect)

☐ to assess the effect of *adhering to intervention* (the 'per-protocol' effect)

If the aim is to assess the effect of *adhering to intervention*, select the deviations from intended intervention that should be addressed (at least one must be checked):

☐ occurrence of non-protocol interventions

☐ failures in implementing the intervention that could have affected the outcome

☐ non-adherence to their assigned intervention by trial participants

Which of the following sources were <u>obtained</u> to help inform the risk-of-bias assessment? (tick as many as apply)

X Journal article(s) with results of the trial

☐ Trial protocol

☐ Statistical analysis plan (SAP)

☐ Non-commercial trial registry record (e.g. ClinicalTrials.gov record)

☐ Company-owned trial registry record (e.g. GSK Clinical Study Register record)

☐ "Grey literature" (e.g. unpublished thesis)

☐ Conference abstract(s) about the trial

☐ Regulatory document (e.g. Clinical Study Report, Drug Approval Package)

☐ Research ethics application

☐ Grant database summary (e.g. NIH RePORTER or Research Councils UK Gateway to Research)

☐ Personal communication with trialist

☐ Personal communication with the sponsor

Risk of bias assessment

Responses underlined in green are potential markers for low risk of bias, and responses in red are potential markers for a risk of bias. Where questions relate only to sign posts to other questions, no formatting is used.

Domain 1: Risk of bias arising from the randomization process

Signalling questions	Comments	Response options
**1.1 Was the allocation sequence random?**	Y	Y / PY / PN / N / NI
**1.2 Was the allocation sequence concealed until participants were enrolled and assigned to interventions?**	PY	Y / PY / PN / N / NI
**1.3 Did baseline differences between intervention groups suggest a problem with the randomization process?**	PN	Y / PY / PN / N / NI
**Risk-of-bias judgement**	Low	Low / High / Some concerns
Optional: What is the predicted direction of bias arising from the randomization process?		NA / Favours experimental / Favours comparator / Towards null /Away from null / Unpredictable

Domain 2: Risk of bias due to deviations from the intended interventions (*effect of assignment to intervention*)

Signalling questions	Comments	Response options
**2.1. Were participants aware of their assigned intervention during the trial?**	Y	Y / PY / PN / N / NI
**2.2. Were carers and people delivering the interventions aware of participants' assigned intervention during the trial?**	Y	Y / PY / PN / N / NI
**2.3. If Y/PY/NI to 2.1 or 2.2: Were there deviations from the intended intervention that arose because of the trial context?**	PN	NA / Y / PY / PN / N / NI
**2.4 If Y/PY to 2.3: Were these deviations likely to have affected the outcome?**		NA / Y / PY / PN / N / NI
**2.5. If Y/PY/NI to 2.4: Were these deviations from intended intervention balanced between groups?**		NA / Y / PY / PN / N / NI
**2.6 Was an appropriate analysis used to estimate the effect of assignment to intervention?**	Y	Y / PY / PN / N / NI
**2.7 If N/PN/NI to 2.6: Was there potential for a substantial impact (on the result) of the failure to analyse participants in the group to which they were randomized?**		NA / Y / PY / PN / N / NI
**Risk-of-bias judgement**	low	Low / High / Some concerns

Optional: What is the predicted direction of bias due to deviations from intended interventions?		NA / Favours experimental / Favours comparator / Towards null /Away from null / Unpredictable

Domain 3: Missing outcome data

Signalling questions	Comments	Response options
**3.1 Were data for this outcome available for all, or nearly all, participants randomized?**	N	Y / PY / PN / N / NI
**3.2 If N/PN/NI to 3.1: Is there evidence that the result was not biased by missing outcome data?**	PN	NA / Y / PY / PN / N
**3.3 If N/PN to 3.2: Could missingness in the outcome depend on its true value?**	PN	NA / Y / PY / PN / N / NI
**3.4 If Y/PY/NI to 3.3: Is it likely that missingness in the outcome depended on its true value?**		NA / Y / PY / PN / N / NI
**Risk-of-bias judgement**	Low	Low / High / Some concerns
Optional: What is the predicted direction of bias due to missing outcome data?		NA / Favours experimental / Favours comparator / Towards null /Away from null / Unpredictable

Domain 4: Risk of bias in measurement of the outcome

Signalling questions	Comments	Response options
**4.1 Was the method of measuring the outcome inappropriate?**	N	Y / PY / PN / N / NI
**4.2 Could measurement or ascertainment of the outcome have differed between intervention groups?**	N	Y / PY / PN / N / NI
**4.3 If N/PN/NI to 4.1 and 4.2: Were outcome assessors aware of the intervention received by study participants?**	Y	NA / Y / PY / PN / N / NI
**4.4 If Y/PY/NI to 4.3: Could assessment of the outcome have been influenced by knowledge of intervention received?**	PN	NA / Y / PY / PN / N / NI
**4.5 If Y/PY/NI to 4.4: Is it likely that assessment of the outcome was influenced by knowledge of intervention received?**		NA / Y / PY / PN / N / NI
**Risk-of-bias judgement**	Low	Low / High / Some concerns
Optional: What is the predicted direction of bias in measurement of the outcome?		NA / Favours experimental / Favours comparator / Towards null /Away from null / Unpredictable

Domain 5: Risk of bias in selection of the reported result

Signalling questions	Comments	Response options
**5.1 Were the data that produced this result analysed in accordance with a pre-specified analysis plan that was finalized before unblinded outcome data were available for analysis?**	Y	Y / PY / PN / N / NI
**Is the numerical result being assessed likely to have been selected, on the basis of the results, from...**		
**5.2. ... multiple eligible outcome measurements (e.g. scales, definitions, time points) within the outcome domain?**	N	Y / PY / PN / N / NI
**5.3 ... multiple eligible analyses of the data?**	N	Y / PY / PN / N / NI
**Risk-of-bias judgement**	Low	Low / High / Some concerns
Optional: What is the predicted direction of bias due to selection of the reported result?		NA / Favours experimental / Favours comparator / Towards null /Away from null / Unpredictable

Overall risk of bias

**Risk-of-bias judgement**	Low	Low / High / Some concerns
Optional: What is the overall predicted direction of bias for this outcome?		NA / Favours experimental / Favours comparator / Towards null /Away from null / Unpredictable
Comments		

Brunkhorst, 2012

Study details

Reference

Brunkhorst FM et al. Effect of Empirical Treatment With Moxifloxacin and Meropenem vs Meropenem on Sepsis-Related Organ Dysfunction in Patients With Severe Sepsis. A Randomized Trial. JAMA, June 13, 2012—Vol 307, No. 22

Study design

X Individually-randomized parallel-group trial

☐ Cluster-randomized parallel-group trial

☐ Individually randomized cross-over (or other matched) trial

For the purposes of this assessment, the interventions being compared are defined as

Experimental: Moxifloxacin + Meropenem

Comparator: Meropenem

Specify which outcome is being assessed for risk of bias

28- and 90-day mortality

Specify the numerical result being assessed. In case of multiple alternative analyses being presented, specify the numeric result (e.g. RR = 1.52 (95% CI 0.83 to 2.77) and/or a reference (e.g. to a table, figure or paragraph) that uniquely defines the result being assessed.

28-day mortality: 23.9% vs. 21.9%

90-day mortality: 35.3%. vs 32.1%

Is the review team's aim for this result…?

X to assess the effect of *assignment to intervention* (the 'intention-to-treat' effect)

☐ to assess the effect of *adhering to intervention* (the 'per-protocol' effect)

If the aim is to assess the effect of *adhering to intervention*, select the deviations from intended intervention that should be addressed (at least one must be checked):

☐ occurrence of non-protocol interventions

☐ failures in implementing the intervention that could have affected the outcome

☐ non-adherence to their assigned intervention by trial participants

Which of the following sources were obtained to help inform the risk-of-bias assessment? (tick as many as apply)

☐ Journal article(s) with results of the trial

☐ Trial protocol

☐ Statistical analysis plan (SAP)

X Non-commercial trial registry record (e.g. ClinicalTrials.gov record)

☐ Company-owned trial registry record (e.g. GSK Clinical Study Register record)

☐ "Grey literature" (e.g. unpublished thesis)

☐ Conference abstract(s) about the trial

☐ Regulatory document (e.g. Clinical Study Report, Drug Approval Package)

☐ Research ethics application

☐ Grant database summary (e.g. NIH RePORTER or Research Councils UK Gateway to Research)

☐ Personal communication with trialist

☐ Personal communication with the sponsor

Risk of bias assessment

Responses underlined in green are potential markers for low risk of bias, and responses in red are potential markers for a risk of bias. Where questions relate only to sign posts to other questions, no formatting is used.

Domain 1: Risk of bias arising from the randomization process

Signalling questions	Comments	Response options
**1.1 Was the allocation sequence random?**	Y	Y / PY / PN / N / NI
**1.2 Was the allocation sequence concealed until participants were enrolled and assigned to interventions?**	PY	Y / PY / PN / N / NI
**1.3 Did baseline differences between intervention groups suggest a problem with the randomization process?**	N	Y / PY / PN / N / NI
**Risk-of-bias judgement**	Low	Low / High / Some concerns
Optional: What is the predicted direction of bias arising from the randomization process?		NA / Favours experimental / Favours comparator / Towards null /Away from null / Unpredictable

Domain 2: Risk of bias due to deviations from the intended interventions (*effect of assignment to intervention*)

**Signalling questions**	**Comments**	**Response options**
**2.1. Were participants aware of their assigned intervention during the trial?**	Y /	Y / PY / PN / N / NI
**2.2. Were carers and people delivering the interventions aware of participants' assigned intervention during the trial?**	Y	Y / PY / PN / N / NI
**2.3. If Y/PY/NI to 2.1 or 2.2: Were there deviations from the intended intervention that arose because of the trial context?**	N	NA / Y / PY / PN / N / NI
**2.4 If Y/PY to 2.3: Were these deviations likely to have affected the outcome?**		NA / Y / PY / PN / N / NI
**2.5. If Y/PY/NI to 2.4: Were these deviations from intended intervention balanced between groups?**		NA / Y / PY / PN / N / NI
**2.6 Was an appropriate analysis used to estimate the effect of assignment to intervention?**	Y	Y / PY / PN / N / NI
**2.7 If N/PN/NI to 2.6: Was there potential for a substantial impact (on the result) of the failure to analyse participants in the group to which they were randomized?**		NA / Y / PY / PN / N / NI
**Risk-of-bias judgement**	Low	Low / High / Some concerns

Optional: What is the predicted direction of bias due to deviations from intended interventions?		NA / Favours experimental / Favours comparator / Towards null /Away from null / Unpredictable

Domain 3: Missing outcome data

Signalling questions	Comments	Response options
**3.1 Were data for this outcome available for all, or nearly all, participants randomized?**	Y	Y / PY / PN / N / NI
**3.2 If N/PN/NI to 3.1: Is there evidence that the result was not biased by missing outcome data?**		NA / Y / PY / PN / N
**3.3 If N/PN to 3.2: Could missingness in the outcome depend on its true value?**		NA / Y / PY / PN / N / NI
**3.4 If Y/PY/NI to 3.3: Is it likely that missingness in the outcome depended on its true value?**		NA / Y / PY / PN / N / NI
**Risk-of-bias judgement**	Low risk	Low / High / Some concerns
Optional: What is the predicted direction of bias due to missing outcome data?		NA / Favours experimental / Favours comparator / Towards null /Away from null / Unpredictable

Domain 4: Risk of bias in measurement of the outcome

Signalling questions	Comments	Response options
**4.1 Was the method of measuring the outcome inappropriate?**	N	Y / PY / PN / N / NI
**4.2 Could measurement or ascertainment of the outcome have differed between intervention groups?**	N	Y / PY / PN / N / NI
**4.3 If N/PN/NI to 4.1 and 4.2: Were outcome assessors aware of the intervention received by study participants?**	Y	NA / Y / PY / PN / N / NI
**4.4 If Y/PY/NI to 4.3: Could assessment of the outcome have been influenced by knowledge of intervention received?**	N	NA / Y / PY / PN / N / NI
**4.5 If Y/PY/NI to 4.4: Is it likely that assessment of the outcome was influenced by knowledge of intervention received?**		NA / Y / PY / PN / N / NI
**Risk-of-bias judgement**	Low	Low / High / Some concerns
Optional: What is the predicted direction of bias in measurement of the outcome?		NA / Favours experimental / Favours comparator / Towards null /Away from null / Unpredictable

Domain 5: Risk of bias in selection of the reported result

Signalling questions	Comments	Response options
**5.1 Were the data that produced this result analysed in accordance with a pre-specified analysis plan that was finalized before unblinded outcome data were available for analysis?**	Y	Y / PY / PN / N / NI
**Is the numerical result being assessed likely to have been selected, on the basis of the results, from...**		
**5.2. ... multiple eligible outcome measurements (e.g. scales, definitions, time points) within the outcome domain?**	N	Y / PY / PN / N / NI
**5.3 ... multiple eligible analyses of the data?**	N	Y / PY / PN / N / NI
**Risk-of-bias judgement**	Low	Low / High / Some concerns
Optional: What is the predicted direction of bias due to selection of the reported result?		NA / Favours experimental / Favours comparator / Towards null /Away from null / Unpredictable

Overall risk of bias

**Risk-of-bias judgement**	Low	Low / High / Some concerns
Optional: What is the overall predicted direction of bias for this outcome?		NA / Favours experimental / Favours comparator / Towards null /Away from null / Unpredictable

Elke, 2013

Responses underlined in green are potential markers for low risk of bias, and responses in red are potential markers for a risk of bias. Where questions relate only to sign posts to other questions, no formatting is used.

Signalling questions	Description	Response options
**Bias due to confounding**		
1.1 Is there potential for confounding of the effect of intervention in this study?<br>**If N/PN to 1.1:** the study can be considered to be at low risk of bias due to confounding and no further signalling questions need be considered	Y	Y / PY / PN / N
**If Y/PY to 1.1**: determine whether there is a need to assess time-varying confounding:		
1.2. Was the analysis based on splitting participants' follow up time according to intervention received?<br>**If N/PN**, answer questions relating to baseline confounding (1.4 to 1.6)<br>**If Y/PY**, go to question 1.3.	N	NA / Y / PY / PN / N / NI
1.3. Were intervention discontinuations or switches likely to be related to factors that are prognostic for the outcome?<br>**If N/PN**, answer questions relating to baseline confounding (1.4 to 1.6)<br>**If Y/PY**, answer questions relating to both baseline and time-varying confounding (1.7 and 1.8)		NA / Y / PY / PN / N / NI

**Questions relating to baseline confounding only**		
1.4. Did the authors use an appropriate analysis method that controlled for all the important confounding domains?	PY	NA / Y / PY / PN / N / NI
1.5. **If Y/PY to 1.4**: Were confounding domains that were controlled for measured validly and reliably by the variables available in this study?	Y	NA / Y / PY / PN / N / NI
1.6. Did the authors control for any post-intervention variables that could have been affected by the intervention?	PN	NA / Y / PY / PN / N / NI
**Questions relating to baseline and time-varying confounding**		
1.7. Did the authors use an appropriate analysis method that controlled for all the important confounding domains and for time-varying confounding?	NI	NA / Y / PY / PN / N / NI
1.8. **If Y/PY to 1.7**: Were confounding domains that were controlled for measured validly and reliably by the variables available in this study?		NA / Y / PY / PN / N / NI
**Risk of bias judgement**	Moderate	Low / Moderate / Serious / Critical / NI
Optional: What is the predicted direction of bias due to confounding?		Favours experimental / Favours comparator / Unpredictable

**Bias in selection of participants into the study**		
2.1. Was selection of participants into the study (or into the analysis) based on participant characteristics observed after the start of intervention?<br>**If N/PN to 2.1:** go to 2.4	Py	Y / PY / PN / N / NI
2.2. **If Y/PY to 2.1**: Were the post-intervention variables that influenced selection likely to be associated with intervention?<br>2.3 **If Y/PY to 2.2**: Were the post-intervention variables that influenced selection likely to be influenced by the outcome or a cause of the outcome?	Pn	NA / Y / PY / PN / N / NI<br>NA / Y / PY / PN / N / NI
2.4. Do start of follow-up and start of intervention coincide for most participants?	Y	Y / PY / PN / N / NI
2.5. **If Y/PY to 2.2 and 2.3, or N/PN to 2.4**: Were adjustment techniques used that are likely to correct for the presence of selection biases?		NA / Y / PY / PN / N / NI
**Risk of bias judgement**	Low to Moderate	Low / Moderate / Serious / Critical / NI
Optional: What is the predicted direction of bias due to selection of participants into the study?		Favours experimental / Favours comparator / Towards null /Away from null / Unpredictable

Bias in classification of interventions		
3.1 Were intervention groups clearly defined?	Y	Y / PY / PN / N / NI
3.2 Was the information used to define intervention groups recorded at the start of the intervention?	Y	Y / PY / PN / N / NI
3.3 Could classification of intervention status have been affected by knowledge of the outcome or risk of the outcome?	N	Y / PY / PN / N / NI
**Risk of bias judgement**	Low	Low / Moderate / Serious / Critical / NI
Optional: What is the predicted direction of bias due to classification of interventions?		Favours experimental / Favours comparator / Towards null /Away from null / Unpredictable

Bias due to missing data		
5.1 Were outcome data available for all, or nearly all, participants?	Y	Y / PY / PN / N / NI
5.2 Were participants excluded due to missing data on intervention status?	PN	Y / PY / PN / N / NI
5.3 Were participants excluded due to missing data on other variables needed for the analysis?	PN	Y / PY / PN / N / NI
5.4 **If PN/N to 5.1, or Y/PY to 5.2 or 5.3**: Are the proportion of participants and reasons for missing data similar across interventions?		NA / Y / PY / PN / N / NI
5.5 **If PN/N to 5.1, or Y/PY to 5.2 or 5.3**: Is there evidence that results were robust to the presence of missing data?		NA / Y / PY / PN / N / NI
**Risk of bias judgement**	Low	Low / Moderate / Serious / Critical / NI
Optional: What is the predicted direction of bias due to missing data?		Favours experimental / Favours comparator / Towards null /Away from null / Unpredictable

Bias in measurement of outcomes		
6.1 Could the outcome measure have been influenced by knowledge of the intervention received?	N	Y / PY / PN / N / NI
6.2 Were outcome assessors aware of the intervention received by study participants?	PY	Y / PY / PN / N / NI
6.3 Were the methods of outcome assessment comparable across intervention groups?	Y	Y / PY / PN / N / NI
6.4 Were any systematic errors in measurement of the outcome related to intervention received?	N	Y / PY / PN / N / NI
**Risk of bias judgement**	Low	Low / Moderate / Serious / Critical / NI
Optional: What is the predicted direction of bias due to measurement of outcomes?		Favours experimental / Favours comparator / Towards null /Away from null / Unpredictable

**Bias in selection of the reported result**		
Is the reported effect estimate likely to be selected, on the basis of the results, from...		
7.1. ... multiple outcome *measurements* within the outcome domain?	N	Y / PY / PN / N / NI
7.2 ... multiple *analyses* of the intervention-outcome relationship?	PN	Y / PY / PN / N / NI
7.3 ... different *subgroups*?	PY	Y / PY / PN / N / NI
**Risk of bias judgement**	Moderate	Low / Moderate / Serious / Critical / NI
Optional: What is the predicted direction of bias due to selection of the reported result?		Favours experimental / Favours comparator / Towards null /Away from null / Unpredictable

Overall bias		
**Risk of bias judgement**	Low to moderate	Low / Moderate / Serious / Critical / NI
Optional: What is the overall predicted direction of bias for this outcome?		Favours experimental / Favours comparator / Towards null /Away from null / Unpredictable

Kaffarnik, 2013

Responses underlined in green are potential markers for low risk of bias, and responses in red are potential markers for a risk of bias. Where questions relate only to sign posts to other questions, no formatting is used.

Signalling questions	Description	Response options
**Bias due to confounding**		
1.1 Is there potential for confounding of the effect of intervention in this study?<br>**If N/PN to 1.1:** the study can be considered to be at low risk of bias due to confounding and no further signalling questions need be considered	Y	Y / PY / PN / N
**If Y/PY to 1.1**: determine whether there is a need to assess time-varying confounding:		
1.2. Was the analysis based on splitting participants' follow up time according to intervention received?<br>**If N/PN**, answer questions relating to baseline confounding (1.4 to 1.6)<br>**If Y/PY**, go to question 1.3.	N	NA / Y / PY / PN / N / NI
1.3. Were intervention discontinuations or switches likely to be related to factors that are prognostic for the outcome?<br>**If N/PN**, answer questions relating to baseline confounding (1.4 to 1.6)<br>**If Y/PY**, answer questions relating to both baseline and time-varying confounding (1.7 and 1.8)		NA / Y / PY / PN / N / NI

**Questions relating to baseline confounding only**		
1.4. Did the authors use an appropriate analysis method that controlled for all the important confounding domains?	N	NA / Y / PY / PN / N / NI
1.5. **If** **Y/PY** **to 1.4**: Were confounding domains that were controlled for measured validly and reliably by the variables available in this study?		NA / Y / PY / PN / N / NI
1.6. Did the authors control for any post-intervention variables that could have been affected by the intervention?	Y	NA / Y / PY / PN / N / NI
**Questions relating to baseline and time-varying confounding**		
1.7. Did the authors use an appropriate analysis method that controlled for all the important confounding domains and for time-varying confounding?	N	NA / Y / PY / PN / N / NI
1.8. **If** **Y/PY** **to 1.7**: Were confounding domains that were controlled for measured validly and reliably by the variables available in this study?		NA / Y / PY / PN / N / NI
**Risk of bias judgement**	Critical	Low / Moderate / Serious / Critical / NI
Optional: What is the predicted direction of bias due to confounding?		Favours experimental / Favours comparator / Unpredictable

Bias in selection of participants into the study		
2.1. Was selection of participants into the study (or into the analysis) based on participant characteristics observed after the start of intervention?<br>**If N/PN to 2.1:** go to 2.4	N	Y / PY / PN / N / NI
2.2. **If Y/PY to 2.1**: Were the post-intervention variables that influenced selection likely to be associated with intervention?		NA / Y / PY / PN / N / NI
2.3 **If Y/PY to 2.2**: Were the post-intervention variables that influenced selection likely to be influenced by the outcome or a cause of the outcome?		NA / Y / PY / PN / N / NI
2.4. Do start of follow-up and start of intervention coincide for most participants?	Y	Y / PY / PN / N / NI
2.5. **If Y/PY to 2.2 and 2.3, or N/PN to 2.4**: Were adjustment techniques used that are likely to correct for the presence of selection biases?		NA / Y / PY / PN / N / NI
**Risk of bias judgement**	Low	Low / Moderate / Serious / Critical / NI
Optional: What is the predicted direction of bias due to selection of participants into the study?		Favours experimental / Favours comparator / Towards null /Away from null / Unpredictable

**Bias in classification of interventions**		
3.1 Were intervention groups clearly defined?	Y	Y / PY / PN / N / NI
3.2 Was the information used to define intervention groups recorded at the start of the intervention?	Y	Y / PY / PN / N / NI
3.3 Could classification of intervention status have been affected by knowledge of the outcome or risk of the outcome?	N	Y / PY / PN / N / NI
**Risk of bias judgement**	Low	Low / Moderate / Serious / Critical / NI
Optional: What is the predicted direction of bias due to classification of interventions?		Favours experimental / Favours comparator / Towards null /Away from null / Unpredictable

Bias due to deviations from intended interventions		
**If your aim for this study is to assess the effect of assignment to intervention, answer questions 4.1 and 4.2**		
4.1. Were there deviations from the intended intervention beyond what would be expected in usual practice?		Y / PY / PN / N / NI
4.2. **If Y/PY to 4.1**: Were these deviations from intended intervention unbalanced between groups *and* likely to have affected the outcome?		NA / Y / PY / PN / N / NI
**If your aim for this study is to assess the effect of starting and adhering to intervention, answer questions 4.3 to 4.6**		
4.3. Were important co-interventions balanced across intervention groups?		Y / PY / PN / N / NI
4.4. Was the intervention implemented successfully for most participants?		Y / PY / PN / N / NI
4.5. Did study participants adhere to the assigned intervention regimen?		Y / PY / PN / N / NI
4.6. **If N/PN to 4.3, 4.4 or 4.5**: Was an appropriate analysis used to estimate the effect of starting and adhering to the intervention?		NA / Y / PY / PN / N / NI
**Risk of bias judgement**		Low / Moderate / Serious / Critical / NI

Optional: What is the predicted direction of bias due to deviations from the intended interventions?		Favours experimental / Favours comparator / Towards null /Away from null / Unpredictable

Bias due to missing data		
5.1 Were outcome data available for all, or nearly all, participants?	Y	Y / PY / PN / N / NI
5.2 Were participants excluded due to missing data on intervention status?	PN	Y / PY / PN / N / NI
5.3 Were participants excluded due to missing data on other variables needed for the analysis?	PN	Y / PY / PN / N / NI
5.4 **If PN/N to 5.1, or Y/PY to 5.2 or 5.3**: Are the proportion of participants and reasons for missing data similar across interventions?		NA / Y / PY / PN / N / NI
5.5 **If PN/N to 5.1, or Y/PY to 5.2 or 5.3**: Is there evidence that results were robust to the presence of missing data?		NA / Y / PY / PN / N / NI
**Risk of bias judgement**	Low	Low / Moderate / Serious / Critical / NI
Optional: What is the predicted direction of bias due to missing data?		Favours experimental / Favours comparator / Towards null /Away from null / Unpredictable

**Bias in measurement of outcomes**		
6.1 Could the outcome measure have been influenced by knowledge of the intervention received?	N	Y / PY / PN / N / NI
6.2 Were outcome assessors aware of the intervention received by study participants?	Y	Y / PY / PN / N / NI
6.3 Were the methods of outcome assessment comparable across intervention groups?	Y	Y / PY / PN / N / NI
6.4 Were any systematic errors in measurement of the outcome related to intervention received?	PN	Y / PY / PN / N / NI
**Risk of bias judgement**	Moderate	Low / Moderate / Serious / Critical / NI
Optional: What is the predicted direction of bias due to measurement of outcomes?		Favours experimental / Favours comparator / Towards null /Away from null / Unpredictable

Bias in selection of the reported result		
Is the reported effect estimate likely to be selected, on the basis of the results, from...		
7.1. ... multiple outcome *measurements* within the outcome domain?	PY	Y / PY / PN / N / NI
7.2 ... multiple *analyses* of the intervention-outcome relationship?	PN	Y / PY / PN / N / NI
7.3 ... different *subgroups*?	PY	Y / PY / PN / N / NI
**Risk of bias judgement**	Serious	Low / Moderate / Serious / Critical / NI
Optional: What is the predicted direction of bias due to selection of the reported result?		Favours experimental / Favours comparator / Towards null /Away from null / Unpredictable

Overall bias		
**Risk of bias judgement**	Serious	Low / Moderate / Serious / Critical / NI
Optional: What is the overall predicted direction of bias for this outcome?		Favours experimental / Favours comparator / Towards null /Away from null / Unpredictable

Keh, 2016

Study details

**Reference**	Keh D, Trips E, Marx G, et al. Effect of Hydrocortisone on Development of Shock Among Patients With Severe Sepsis: The HYPRESS Randomized Clinical Trial. JAMA. 2016;316(17):1775–1785.

Study design

X Individually-randomized parallel-group trial

☐ Cluster-randomized parallel-group trial

☐ Individually randomized cross-over (or other matched) trial

For the purposes of this assessment, the interventions being compared are defined as

Experimental: Hydrocortisone Comparator: Placebo

**Specify which outcome is being assessed for risk of bias**	28-day mortality<br>90-day mortality
**Specify the numerical result being assessed.** In case of multiple alternative analyses being presented, specify the numeric result (e.g. RR = 1.52 (95% CI 0.83 to 2.77) and/or a reference (e.g. to a table, figure or paragraph) that uniquely defines the result being assessed.	28-day: 8.8% (experimental) vs 8.2% (comparator)<br>90-day: 19.9% (experimental) vs 16.7% (comparator)

Is the review team's aim for this result…?

X to assess the effect of *assignment to intervention* (the 'intention-to-treat' effect)

☐ to assess the effect of *adhering to intervention* (the 'per-protocol' effect)

If the aim is to assess the effect of *adhering to intervention*, select the deviations from intended intervention that should be addressed (at least one must be checked):

☐ occurrence of non-protocol interventions

☐ failures in implementing the intervention that could have affected the outcome

☐ non-adherence to their assigned intervention by trial participants

Which of the following sources were <u>obtained</u> to help inform the risk-of-bias assessment? (tick as many as apply)

☐ Journal article(s) with results of the trial

X Trial protocol

X Statistical analysis plan (SAP)

X Non-commercial trial registry record (e.g. ClinicalTrials.gov record)

☐ Company-owned trial registry record (e.g. GSK Clinical Study Register record)

☐ “Grey literature” (e.g. unpublished thesis)

☐ Conference abstract(s) about the trial

☐ Regulatory document (e.g. Clinical Study Report, Drug Approval Package)

☐ Research ethics application

☐ Grant database summary (e.g. NIH RePORTER or Research Councils UK Gateway to Research)

☐ Personal communication with trialist

☐ Personal communication with the sponsor

Risk of bias assessment

Responses underlined in green are potential markers for low risk of bias, and responses in red are potential markers for a risk of bias. Where questions relate only to sign posts to other questions, no formatting is used.

Domain 1: Risk of bias arising from the randomization process

Signalling questions	Comments	Response options
**1.1 Was the allocation sequence random?**	Y	Y / PY / PN / N / NI
**1.2 Was the allocation sequence concealed until participants were enrolled and assigned to interventions?**	PY	Y / PY / PN / N / NI
**1.3 Did baseline differences between intervention groups suggest a problem with the randomization process?**	N	Y / PY / PN / N / NI
**Risk-of-bias judgement**	Low	Low / High / Some concerns
Optional: What is the predicted direction of bias arising from the randomization process?		NA / Favours experimental / Favours comparator / Towards null /Away from null / Unpredictable

Domain 2: Risk of bias due to deviations from the intended interventions (*effect of assignment to intervention*)

Signalling questions	Comments	Response options
**2.1. Were participants aware of their assigned intervention during the trial?**	N	Y / PY / PN / N / NI
**2.2. Were carers and people delivering the interventions aware of participants' assigned intervention during the trial?**	N	Y / PY / PN / N / NI
**2.3. If Y/PY/NI to 2.1 or 2.2: Were there deviations from the intended intervention that arose because of the trial context?**		NA / Y / PY / PN / N / NI
**2.4 If Y/PY to 2.3: Were these deviations likely to have affected the outcome?**		NA / Y / PY / PN / N / NI
**2.5. If Y/PY/NI to 2.4: Were these deviations from intended intervention balanced between groups?**		NA / Y / PY / PN / N / NI
**2.6 Was an appropriate analysis used to estimate the effect of assignment to intervention?**	Y	Y / PY / PN / N / NI
**2.7 If N/PN/NI to 2.6: Was there potential for a substantial impact (on the result) of the failure to analyse participants in the group to which they were randomized?**		NA / Y / PY / PN / N / NI
**Risk-of-bias judgement**	Low	Low / High / Some concerns

Optional: What is the predicted direction of bias due to deviations from intended interventions?		NA / Favours experimental / Favours comparator / Towards null /Away from null / Unpredictable

Domain 3: Missing outcome data

Signalling questions	Comments	Response options
**3.1 Were data for this outcome available for all, or nearly all, participants randomized?**	Y	Y / PY / PN / N / NI
**3.2 If N/PN/NI to 3.1: Is there evidence that the result was not biased by missing outcome data?**		NA / Y / PY / PN / N
**3.3 If N/PN to 3.2: Could missingness in the outcome depend on its true value?**		NA / Y / PY / PN / N / NI
**3.4 If Y/PY/NI to 3.3: Is it likely that missingness in the outcome depended on its true value?**		NA / Y / PY / PN / N / NI
**Risk-of-bias judgement**	Low	Low / High / Some concerns
Optional: What is the predicted direction of bias due to missing outcome data?		NA / Favours experimental / Favours comparator / Towards null /Away from null / Unpredictable

Domain 4: Risk of bias in measurement of the outcome

Signalling questions	Comments	Response options
**4.1 Was the method of measuring the outcome inappropriate?**	N	Y / PY / PN / N / NI
**4.2 Could measurement or ascertainment of the outcome have differed between intervention groups?**	N	Y / PY / PN / N / NI
**4.3 If N/PN/NI to 4.1 and 4.2: Were outcome assessors aware of the intervention received by study participants?**	Y	NA / Y / PY / PN / N / NI
**4.4 If Y/PY/NI to 4.3: Could assessment of the outcome have been influenced by knowledge of intervention received?**		NA / Y / PY / PN / N / NI
**4.5 If Y/PY/NI to 4.4: Is it likely that assessment of the outcome was influenced by knowledge of intervention received?**		NA / Y / PY / PN / N / NI
**Risk-of-bias judgement**	Low	Low / High / Some concerns
Optional: What is the predicted direction of bias in measurement of the outcome?		NA / Favours experimental / Favours comparator / Towards null /Away from null / Unpredictable

Domain 5: Risk of bias in selection of the reported result

Signalling questions	Comments	Response options
**5.1 Were the data that produced this result analysed in accordance with a pre-specified analysis plan that was finalized before unblinded outcome data were available for analysis?**	Y	Y / PY / PN / N / NI
**Is the numerical result being assessed likely to have been selected, on the basis of the results, from...**		
**5.2. ... multiple eligible outcome measurements (e.g. scales, definitions, time points) within the outcome domain?**	N	Y / PY / PN / N / NI
**5.3 ... multiple eligible analyses of the data?**	PN	Y / PY / PN / N / NI
**Risk-of-bias judgement**	Low	Low / High / Some concerns
Optional: What is the predicted direction of bias due to selection of the reported result?		NA / Favours experimental / Favours comparator / Towards null /Away from null / Unpredictable

Overall risk of bias

**Risk-of-bias judgement**	Low	Low / High / Some concerns
Optional: What is the overall predicted direction of bias for this outcome?		NA / Favours experimental / Favours comparator / Towards null /Away from null / Unpredictable

Koch, 2010

Responses underlined in green are potential markers for low risk of bias, and responses in red are potential markers for a risk of bias. Where questions relate only to sign posts to other questions, no formatting is used.

Signalling questions	Description	Response options
**Bias due to confounding**		
1.1 Is there potential for confounding of the effect of intervention in this study?<br>**If N/PN to 1.1:** the study can be considered to be at low risk of bias due to confounding and no further signalling questions need be considered	Y	Y / PY / PN / N
**If Y/PY to 1.1**: determine whether there is a need to assess time-varying confounding:		
1.2. Was the analysis based on splitting participants' follow up time according to intervention received?<br>**If N/PN**, answer questions relating to baseline confounding (1.4 to 1.6)<br>**If Y/PY**, go to question 1.3.	N	NA / Y / PY / PN / N / NI
1.3. Were intervention discontinuations or switches likely to be related to factors that are prognostic for the outcome?<br>**If N/PN**, answer questions relating to baseline confounding (1.4 to 1.6)<br>**If Y/PY**, answer questions relating to both baseline and time-varying confounding (1.7 and 1.8)		NA / Y / PY / PN / N / NI

**Questions relating to baseline confounding only**		
1.4. Did the authors use an appropriate analysis method that controlled for all the important confounding domains?	N	NA / Y / PY / PN / N / NI
1.5. **If Y/PY to 1.4**: Were confounding domains that were controlled for measured validly and reliably by the variables available in this study?		NA / Y / PY / PN / N / NI
1.6. Did the authors control for any post-intervention variables that could have been affected by the intervention?	N	NA / Y / PY / PN / N / NI
**Questions relating to baseline and time-varying confounding**		
1.7. Did the authors use an appropriate analysis method that controlled for all the important confounding domains and for time-varying confounding?	N	NA / Y / PY / PN / N / NI
1.8. **If Y/PY to 1.7**: Were confounding domains that were controlled for measured validly and reliably by the variables available in this study?		NA / Y / PY / PN / N / NI
**Risk of bias judgement**	Serious	Low / Moderate / Serious / Critical / NI
Optional: What is the predicted direction of bias due to confounding?		Favours experimental / Favours comparator / Unpredictable

Bias in selection of participants into the study		
2.1. Was selection of participants into the study (or into the analysis) based on participant characteristics observed after the start of intervention?<br>**If N/PN to 2.1:** go to 2.4	N	Y / PY / PN / N / NI
2.2. **If Y/PY to 2.1**: Were the post-intervention variables that influenced selection likely to be associated with intervention?		NA / Y / PY / PN / N / NI
2.3 **If Y/PY to 2.2**: Were the post-intervention variables that influenced selection likely to be influenced by the outcome or a cause of the outcome?		NA / Y / PY / PN / N / NI
2.4. Do start of follow-up and start of intervention coincide for most participants?	Y	Y / PY / PN / N / NI
2.5. **If Y/PY to 2.2 and 2.3, or N/PN to 2.4**: Were adjustment techniques used that are likely to correct for the presence of selection biases?		NA / Y / PY / PN / N / NI
**Risk of bias judgement**	Low	Low / Moderate / Serious / Critical / NI
Optional: What is the predicted direction of bias due to selection of participants into the study?		Favours experimental / Favours comparator / Towards null /Away from null / Unpredictable

Bias in classification of interventions		
3.1 Were intervention groups clearly defined?	Y	Y / PY / PN / N / NI
3.2 Was the information used to define intervention groups recorded at the start of the intervention?	Y	Y / PY / PN / N / NI
3.3 Could classification of intervention status have been affected by knowledge of the outcome or risk of the outcome?	PN	Y / PY / PN / N / NI
**Risk of bias judgement**	Low	Low / Moderate / Serious / Critical / NI
Optional: What is the predicted direction of bias due to classification of interventions?		Favours experimental / Favours comparator / Towards null /Away from null / Unpredictable

Bias due to deviations from intended interventions		
**If your aim for this study is to assess the effect of assignment to intervention, answer questions 4.1 and 4.2**		
4.1. Were there deviations from the intended intervention beyond what would be expected in usual practice?		Y / PY / PN / N / NI
4.2. **If Y/PY to 4.1**: Were these deviations from intended intervention unbalanced between groups *and* likely to have affected the outcome?		NA / Y / PY / PN / N / NI
**If your aim for this study is to assess the effect of starting and adhering to intervention, answer questions 4.3 to 4.6**		
4.3. Were important co-interventions balanced across intervention groups?		Y / PY / PN / N / NI
4.4. Was the intervention implemented successfully for most participants?		Y / PY / PN / N / NI
4.5. Did study participants adhere to the assigned intervention regimen?		Y / PY / PN / N / NI
4.6. **If N/PN to 4.3, 4.4 or 4.5**: Was an appropriate analysis used to estimate the effect of starting and adhering to the intervention?		NA / Y / PY / PN / N / NI
**Risk of bias judgement**		Low / Moderate / Serious / Critical / NI

Optional: What is the predicted direction of bias due to deviations from the intended interventions?		Favours experimental / Favours comparator / Towards null /Away from null / Unpredictable

Bias due to missing data		
5.1 Were outcome data available for all, or nearly all, participants?	PY	Y / PY / PN / N / NI
5.2 Were participants excluded due to missing data on intervention status?	PN	Y / PY / PN / N / NI
5.3 Were participants excluded due to missing data on other variables needed for the analysis?	PN	Y / PY / PN / N / NI
5.4 **If PN/N to 5.1, or Y/PY to 5.2 or 5.3**: Are the proportion of participants and reasons for missing data similar across interventions?		NA / Y / PY / PN / N / NI
5.5 **If PN/N to 5.1, or Y/PY to 5.2 or 5.3**: Is there evidence that results were robust to the presence of missing data?		NA / Y / PY / PN / N / NI
**Risk of bias judgement**	Moderate	Low / Moderate / Serious / Critical / NI
Optional: What is the predicted direction of bias due to missing data?		Favours experimental / Favours comparator / Towards null /Away from null / Unpredictable

Bias in measurement of outcomes		
6.1 Could the outcome measure have been influenced by knowledge of the intervention received?	N	Y / PY / PN / N / NI
6.2 Were outcome assessors aware of the intervention received by study participants?	Y	Y / PY / PN / N / NI
6.3 Were the methods of outcome assessment comparable across intervention groups?	Y	Y / PY / PN / N / NI
6.4 Were any systematic errors in measurement of the outcome related to intervention received?	N	Y / PY / PN / N / NI
**Risk of bias judgement**	Low	Low / Moderate / Serious / Critical / NI
Optional: What is the predicted direction of bias due to measurement of outcomes?		Favours experimental / Favours comparator / Towards null /Away from null / Unpredictable

Bias in selection of the reported result		
Is the reported effect estimate likely to be selected, on the basis of the results, from...		
7.1. ... multiple outcome *measurements* within the outcome domain?	N	Y / PY / PN / N / NI
7.2 ... multiple *analyses* of the intervention-outcome relationship?	N	Y / PY / PN / N / NI
7.3 ... different *subgroups*?	PN	Y / PY / PN / N / NI
**Risk of bias judgement**	Low	Low / Moderate / Serious / Critical / NI
Optional: What is the predicted direction of bias due to selection of the reported result?		Favours experimental / Favours comparator / Towards null /Away from null / Unpredictable

Overall bias		
**Risk of bias judgement**	Low	Low / Moderate / Serious / Critical / NI
Optional: What is the overall predicted direction of bias for this outcome?		Favours experimental / Favours comparator / Towards null /Away from null / Unpredictable

Kristof, 2018

Responses underlined in green are potential markers for low risk of bias, and responses in red are potential markers for a risk of bias. Where questions relate only to sign posts to other questions, no formatting is used.

Signalling questions	Description	Response options
**Bias due to confounding**		
1.1 Is there potential for confounding of the effect of intervention in this study?<br>**If N/PN to 1.1:** the study can be considered to be at low risk of bias due to confounding and no further signalling questions need be considered	Y	Y / PY / PN / N
**If Y/PY to 1.1**: determine whether there is a need to assess time-varying confounding:		
1.2. Was the analysis based on splitting participants' follow up time according to intervention received?<br>**If N/PN**, answer questions relating to baseline confounding (1.4 to 1.6)<br>**If Y/PY**, go to question 1.3.	N	NA / Y / PY / PN / N / NI
1.3. Were intervention discontinuations or switches likely to be related to factors that are prognostic for the outcome?<br>**If N/PN**, answer questions relating to baseline confounding (1.4 to 1.6)<br>**If Y/PY**, answer questions relating to both baseline and time-varying confounding (1.7 and 1.8)		NA / Y / PY / PN / N / NI

**Questions relating to baseline confounding only**		
1.4. Did the authors use an appropriate analysis method that controlled for all the important confounding domains?	PY	NA / Y / PY / PN / N / NI
1.5. **If Y/PY to 1.4**: Were confounding domains that were controlled for measured validly and reliably by the variables available in this study?	PY	NA / Y / PY / PN / N / NI
1.6. Did the authors control for any post-intervention variables that could have been affected by the intervention?	N	NA / Y / PY / PN / N / NI
**Questions relating to baseline and time-varying confounding**		
1.7. Did the authors use an appropriate analysis method that controlled for all the important confounding domains and for time-varying confounding?	PY	NA / Y / PY / PN / N / NI
1.8. **If Y/PY to 1.7**: Were confounding domains that were controlled for measured validly and reliably by the variables available in this study?	Y	NA / Y / PY / PN / N / NI
**Risk of bias judgement**	Moderate	Low / Moderate / Serious / Critical / NI
Optional: What is the predicted direction of bias due to confounding?		Favours experimental / Favours comparator / Unpredictable

Bias in selection of participants into the study		
2.1. Was selection of participants into the study (or into the analysis) based on participant characteristics observed after the start of intervention?<br>**If N/PN to 2.1:** go to 2.4<br>2.2. **If Y/PY to 2.1**: Were the post-intervention variables that influenced selection likely to be associated with intervention?<br>2.3 **If Y/PY to 2.2**: Were the post-intervention variables that influenced selection likely to be influenced by the outcome or a cause of the outcome?	N	Y / PY / PN / N / NI<br><br>NA / Y / PY / PN / N / NI<br><br>NA / Y / PY / PN / N / NI
2.4. Do start of follow-up and start of intervention coincide for most participants?	Y	Y / PY / PN / N / NI
2.5. **If Y/PY to 2.2 and 2.3, or N/PN to 2.4**: Were adjustment techniques used that are likely to correct for the presence of selection biases?		NA / Y / PY / PN / N / NI
**Risk of bias judgement**	Low	Low / Moderate / Serious / Critical / NI
Optional: What is the predicted direction of bias due to selection of participants into the study?		Favours experimental / Favours comparator / Towards null /Away from null / Unpredictable

Bias in classification of interventions		
3.1 Were intervention groups clearly defined?	Y	Y / PY / PN / N / NI
3.2 Was the information used to define intervention groups recorded at the start of the intervention?	Y	Y / PY / PN / N / NI
3.3 Could classification of intervention status have been affected by knowledge of the outcome or risk of the outcome?	N	Y / PY / PN / N / NI
**Risk of bias judgement**	Low	Low / Moderate / Serious / Critical / NI
Optional: What is the predicted direction of bias due to classification of interventions?		Favours experimental / Favours comparator / Towards null /Away from null / Unpredictable

Bias due to deviations from intended interventions		
**If your aim for this study is to assess the effect of assignment to intervention, answer questions 4.1 and 4.2**		
4.1. Were there deviations from the intended intervention beyond what would be expected in usual practice?		Y / PY / PN / N / NI
4.2. **If Y/PY to 4.1**: Were these deviations from intended intervention unbalanced between groups *and* likely to have affected the outcome?		NA / Y / PY / PN / N / NI
**If your aim for this study is to assess the effect of starting and adhering to intervention, answer questions 4.3 to 4.6**		
4.3. Were important co-interventions balanced across intervention groups?		Y / PY / PN / N / NI
4.4. Was the intervention implemented successfully for most participants?		Y / PY / PN / N / NI
4.5. Did study participants adhere to the assigned intervention regimen?		Y / PY / PN / N / NI
4.6. **If N/PN to 4.3, 4.4 or 4.5**: Was an appropriate analysis used to estimate the effect of starting and adhering to the intervention?		NA / Y / PY / PN / N / NI
**Risk of bias judgement**		Low / Moderate / Serious / Critical / NI

Optional: What is the predicted direction of bias due to deviations from the intended interventions?		Favours experimental / Favours comparator / Towards null /Away from null / Unpredictable

**Bias due to missing data**		
5.1 Were outcome data available for all, or nearly all, participants?	PY	Y / PY / PN / N / NI
5.2 Were participants excluded due to missing data on intervention status?	PN	Y / PY / PN / N / NI
5.3 Were participants excluded due to missing data on other variables needed for the analysis?	PN	Y / PY / PN / N / NI
5.4 **If PN/N to 5.1, or Y/PY to 5.2 or 5.3**: Are the proportion of participants and reasons for missing data similar across interventions?		NA / Y / PY / PN / N / NI
5.5 **If PN/N to 5.1, or Y/PY to 5.2 or 5.3**: Is there evidence that results were robust to the presence of missing data?		NA / Y / PY / PN / N / NI
**Risk of bias judgement**	Low	Low / Moderate / Serious / Critical / NI
Optional: What is the predicted direction of bias due to missing data?		Favours experimental / Favours comparator / Towards null /Away from null / Unpredictable

Bias in measurement of outcomes		
6.1 Could the outcome measure have been influenced by knowledge of the intervention received?	N	Y / PY / PN / N / NI
6.2 Were outcome assessors aware of the intervention received by study participants?	Y	Y / PY / PN / N / NI
6.3 Were the methods of outcome assessment comparable across intervention groups?	Y	Y / PY / PN / N / NI
6.4 Were any systematic errors in measurement of the outcome related to intervention received?	N	Y / PY / PN / N / NI
**Risk of bias judgement**	Moderate	Low / Moderate / Serious / Critical / NI
Optional: What is the predicted direction of bias due to measurement of outcomes?		Favours experimental / Favours comparator / Towards null /Away from null / Unpredictable

**Bias in selection of the reported result**		
Is the reported effect estimate likely to be selected, on the basis of the results, from...		
7.1. ... multiple outcome *measurements* within the outcome domain?	N	Y / PY / PN / N / NI
7.2 ... multiple *analyses* of the intervention-outcome relationship?	PN	Y / PY / PN / N / NI
7.3 ... different *subgroups*?	N	Y / PY / PN / N / NI
**Risk of bias judgement**	Low	Low / Moderate / Serious / Critical / NI
Optional: What is the predicted direction of bias due to selection of the reported result?		Favours experimental / Favours comparator / Towards null /Away from null / Unpredictable

Overall bias		
**Risk of bias judgement**	Moderate	Low / Moderate / Serious / Critical / NI
Optional: What is the overall predicted direction of bias for this outcome?		Favours experimental / Favours comparator / Towards null /Away from null / Unpredictable

Mansur, 2015a

Impact of statin therapy on mortality in patients with sepsis-associated acute respiratory distress syndrome (ARDS) depends on ARDS severity: a prospective observational cohort study.

Responses underlined in green are potential markers for low risk of bias, and responses in red are potential markers for a risk of bias. Where questions relate only to sign posts to other questions, no formatting is used.

Signalling questions	Description	Response options
**Bias due to confounding**		
1.1 Is there potential for confounding of the effect of intervention in this study?<br>**If N/PN to 1.1:** the study can be considered to be at low risk of bias due to confounding and no further signalling questions need be considered	PY	Y / PY / PN / N
**If Y/PY to 1.1**: determine whether there is a need to assess time-varying confounding:		
1.2. Was the analysis based on splitting participants' follow up time according to intervention received?<br>**If N/PN**, answer questions relating to baseline confounding (1.4 to 1.6)<br>**If Y/PY**, go to question 1.3.	N	NA / Y / PY / PN / N / NI
1.3. Were intervention discontinuations or switches likely to be related to factors that are prognostic for the outcome?<br>**If N/PN**, answer questions relating to baseline confounding (1.4 to 1.6)<br>**If Y/PY**, answer questions relating to both baseline and time-varying confounding (1.7 and 1.8)		NA / Y / PY / PN / N / NI

**Questions relating to baseline confounding only**		
1.4. Did the authors use an appropriate analysis method that controlled for all the important confounding domains?	Y	NA / Y / PY / PN / N / NI
1.5. **If Y/PY to 1.4**: Were confounding domains that were controlled for measured validly and reliably by the variables available in this study?	Y	NA / Y / PY / PN / N / NI
1.6. Did the authors control for any post-intervention variables that could have been affected by the intervention?	N	NA / Y / PY / PN / N / NI
**Questions relating to baseline and time-varying confounding**		
1.7. Did the authors use an appropriate analysis method that controlled for all the important confounding domains and for time-varying confounding?	PY	NA / Y / PY / PN / N / NI
1.8. **If Y/PY to 1.7**: Were confounding domains that were controlled for measured validly and reliably by the variables available in this study?	Y	NA / Y / PY / PN / N / NI
**Risk of bias judgement**	Low	Low / Moderate / Serious / Critical / NI
Optional: What is the predicted direction of bias due to confounding?		Favours experimental / Favours comparator / Unpredictable

Bias in selection of participants into the study		
2.1. Was selection of participants into the study (or into the analysis) based on participant characteristics observed after the start of intervention?<br>**If N/PN to 2.1:** go to 2.4<br>2.2. **If Y/PY to 2.1**: Were the post-intervention variables that influenced selection likely to be associated with intervention?<br>2.3 **If Y/PY to 2.2**: Were the post-intervention variables that influenced selection likely to be influenced by the outcome or a cause of the outcome?	N	Y / PY / PN / N / NI<br><br>NA / Y / PY / PN / N / NI<br><br>NA / Y / PY / PN / N / NI
2.4. Do start of follow-up and start of intervention coincide for most participants?	Y	Y / PY / PN / N / NI
2.5. **If Y/PY to 2.2 and 2.3, or N/PN to 2.4**: Were adjustment techniques used that are likely to correct for the presence of selection biases?		NA / Y / PY / PN / N / NI
**Risk of bias judgement**	Low	Low / Moderate / Serious / Critical / NI
Optional: What is the predicted direction of bias due to selection of participants into the study?		Favours experimental / Favours comparator / Towards null /Away from null / Unpredictable

**Bias in classification of interventions**		
3.1 Were intervention groups clearly defined?	Y	Y / PY / PN / N / NI
3.2 Was the information used to define intervention groups recorded at the start of the intervention?	Y	Y / PY / PN / N / NI
3.3 Could classification of intervention status have been affected by knowledge of the outcome or risk of the outcome?	Pn	Y / PY / PN / N / NI
**Risk of bias judgement**	Low	Low / Moderate / Serious / Critical / NI
Optional: What is the predicted direction of bias due to classification of interventions?		Favours experimental / Favours comparator / Towards null /Away from null / Unpredictable

Bias due to deviations from intended interventions		
**If your aim for this study is to assess the effect of assignment to intervention, answer questions 4.1 and 4.2**		
4.1. Were there deviations from the intended intervention beyond what would be expected in usual practice?		Y / PY / PN / N / NI
4.2. **If Y/PY to 4.1**: Were these deviations from intended intervention unbalanced between groups *and* likely to have affected the outcome?		NA / Y / PY / PN / N / NI
**If your aim for this study is to assess the effect of starting and adhering to intervention, answer questions 4.3 to 4.6**		
4.3. Were important co-interventions balanced across intervention groups?		Y / PY / PN / N / NI
4.4. Was the intervention implemented successfully for most participants?		Y / PY / PN / N / NI
4.5. Did study participants adhere to the assigned intervention regimen?		Y / PY / PN / N / NI
4.6. **If N/PN to 4.3, 4.4 or 4.5**: Was an appropriate analysis used to estimate the effect of starting and adhering to the intervention?		NA / Y / PY / PN / N / NI
**Risk of bias judgement**		Low / Moderate / Serious / Critical / NI

Optional: What is the predicted direction of bias due to deviations from the intended interventions?		Favours experimental / Favours comparator / Towards null /Away from null / Unpredictable

Bias due to missing data		
5.1 Were outcome data available for all, or nearly all, participants?	Y	Y / PY / PN / N / NI
5.2 Were participants excluded due to missing data on intervention status?	N	Y / PY / PN / N / NI
5.3 Were participants excluded due to missing data on other variables needed for the analysis?	Pn	Y / PY / PN / N / NI
5.4 **If PN/N to 5.1, or Y/PY to 5.2 or 5.3**: Are the proportion of participants and reasons for missing data similar across interventions?		NA / Y / PY / PN / N / NI
5.5 **If PN/N to 5.1, or Y/PY to 5.2 or 5.3**: Is there evidence that results were robust to the presence of missing data?		NA / Y / PY / PN / N / NI
**Risk of bias judgement**	Low	Low / Moderate / Serious / Critical / NI
Optional: What is the predicted direction of bias due to missing data?		Favours experimental / Favours comparator / Towards null /Away from null / Unpredictable

Bias in measurement of outcomes		
6.1 Could the outcome measure have been influenced by knowledge of the intervention received?	N	Y / PY / PN / N / NI
6.2 Were outcome assessors aware of the intervention received by study participants?	Py	Y / PY / PN / N / NI
6.3 Were the methods of outcome assessment comparable across intervention groups?	Y	Y / PY / PN / N / NI
6.4 Were any systematic errors in measurement of the outcome related to intervention received?	N	Y / PY / PN / N / NI
**Risk of bias judgement**	Low	Low / Moderate / Serious / Critical / NI
Optional: What is the predicted direction of bias due to measurement of outcomes?		Favours experimental / Favours comparator / Towards null /Away from null / Unpredictable

Bias in selection of the reported result		
Is the reported effect estimate likely to be selected, on the basis of the results, from...		
7.1. ... multiple outcome *measurements* within the outcome domain?	N	Y / PY / PN / N / NI
7.2 ... multiple *analyses* of the intervention-outcome relationship?	Pn	Y / PY / PN / N / NI
7.3 ... different *subgroups*?	Py	Y / PY / PN / N / NI
**Risk of bias judgement**	Moderate	Low / Moderate / Serious / Critical / NI
Optional: What is the predicted direction of bias due to selection of the reported result?		Favours experimental / Favours comparator / Towards null /Away from null / Unpredictable

Overall bias		
**Risk of bias judgement**	Low	Low / Moderate / Serious / Critical / NI
Optional: What is the overall predicted direction of bias for this outcome?		Favours experimental / Favours comparator / Towards null /Away from null / Unpredictable

Mansur, 2015b

Primary bacteraemia is associated with a higher mortality risk compared with pulmonary and intra-abdominal infections in patients with sepsis: a prospective observational cohort study.

Responses underlined in green are potential markers for low risk of bias, and responses in red are potential markers for a risk of bias. Where questions relate only to sign posts to other questions, no formatting is used.

Signalling questions	Description	Response options
**Bias due to confounding**		
1.1 Is there potential for confounding of the effect of intervention in this study?<br>**If N/PN to 1.1:** the study can be considered to be at low risk of bias due to confounding and no further signalling questions need be considered	Y	Y / PY / PN / N
**If Y/PY to 1.1**: determine whether there is a need to assess time-varying confounding:		
1.2. Was the analysis based on splitting participants' follow up time according to intervention received?<br>**If N/PN**, answer questions relating to baseline confounding (1.4 to 1.6)<br>**If Y/PY**, go to question 1.3.	N	NA / Y / PY / PN / N / NI
1.3. Were intervention discontinuations or switches likely to be related to factors that are prognostic for the outcome?<br>**If N/PN**, answer questions relating to baseline confounding (1.4 to 1.6)<br>**If Y/PY**, answer questions relating to both baseline and time-varying confounding (1.7 and 1.8)		NA / Y / PY / PN / N / NI

**Questions relating to baseline confounding only**		
1.4. Did the authors use an appropriate analysis method that controlled for all the important confounding domains?	Y	NA / Y / PY / PN / N / NI
1.5. **If Y/PY to 1.4**: Were confounding domains that were controlled for measured validly and reliably by the variables available in this study?	Y	NA / Y / PY / PN / N / NI
1.6. Did the authors control for any post-intervention variables that could have been affected by the intervention?	N	NA / Y / PY / PN / N / NI
**Questions relating to baseline and time-varying confounding**		
1.7. Did the authors use an appropriate analysis method that controlled for all the important confounding domains and for time-varying confounding?	PN	NA / Y / PY / PN / N / NI
1.8. **If Y/PY to 1.7**: Were confounding domains that were controlled for measured validly and reliably by the variables available in this study?		NA / Y / PY / PN / N / NI
**Risk of bias judgement**	Moderate	Low / Moderate / Serious / Critical / NI
Optional: What is the predicted direction of bias due to confounding?		Favours experimental / Favours comparator / Unpredictable

Bias in selection of participants into the study		
2.1. Was selection of participants into the study (or into the analysis) based on participant characteristics observed after the start of intervention?<br>**If N/PN to 2.1:** go to 2.4<br>2.2. **If Y/PY to 2.1**: Were the post-intervention variables that influenced selection likely to be associated with intervention?<br>2.3 **If Y/PY to 2.2**: Were the post-intervention variables that influenced selection likely to be influenced by the outcome or a cause of the outcome?	N	Y / PY / PN / N / NI<br><br>NA / Y / PY / PN / N / NI<br><br>NA / Y / PY / PN / N / NI
2.4. Do start of follow-up and start of intervention coincide for most participants?	Y	Y / PY / PN / N / NI
2.5. **If Y/PY to 2.2 and 2.3, or N/PN to 2.4**: Were adjustment techniques used that are likely to correct for the presence of selection biases?		NA / Y / PY / PN / N / NI
**Risk of bias judgement**	Low	Low / Moderate / Serious / Critical / NI
Optional: What is the predicted direction of bias due to selection of participants into the study?		Favours experimental / Favours comparator / Towards null /Away from null / Unpredictable

**Bias in classification of interventions**		
3.1 Were intervention groups clearly defined?	Y	Y / PY / PN / N / NI
3.2 Was the information used to define intervention groups recorded at the start of the intervention?	Y	Y / PY / PN / N / NI
3.3 Could classification of intervention status have been affected by knowledge of the outcome or risk of the outcome?	N	Y / PY / PN / N / NI
**Risk of bias judgement**	Low	Low / Moderate / Serious / Critical / NI
Optional: What is the predicted direction of bias due to classification of interventions?		Favours experimental / Favours comparator / Towards null /Away from null / Unpredictable

Bias due to deviations from intended interventions		
**If your aim for this study is to assess the effect of assignment to intervention, answer questions 4.1 and 4.2**		
4.1. Were there deviations from the intended intervention beyond what would be expected in usual practice?		Y / PY / PN / N / NI
4.2. **If Y/PY to 4.1**: Were these deviations from intended intervention unbalanced between groups *and* likely to have affected the outcome?		NA / Y / PY / PN / N / NI
**If your aim for this study is to assess the effect of starting and adhering to intervention, answer questions 4.3 to 4.6**		
4.3. Were important co-interventions balanced across intervention groups?		Y / PY / PN / N / NI
4.4. Was the intervention implemented successfully for most participants?		Y / PY / PN / N / NI
4.5. Did study participants adhere to the assigned intervention regimen?		Y / PY / PN / N / NI
4.6. **If N/PN to 4.3, 4.4 or 4.5**: Was an appropriate analysis used to estimate the effect of starting and adhering to the intervention?		NA / Y / PY / PN / N / NI
**Risk of bias judgement**		Low / Moderate / Serious / Critical / NI

Optional: What is the predicted direction of bias due to deviations from the intended interventions?		Favours experimental / Favours comparator / Towards null /Away from null / Unpredictable

Bias due to missing data		
5.1 Were outcome data available for all, or nearly all, participants?	Y	Y / PY / PN / N / NI
5.2 Were participants excluded due to missing data on intervention status?	N	Y / PY / PN / N / NI
5.3 Were participants excluded due to missing data on other variables needed for the analysis?	PN	Y / PY / PN / N / NI
5.4 **If PN/N to 5.1, or Y/PY to 5.2 or 5.3**: Are the proportion of participants and reasons for missing data similar across interventions?		NA / Y / PY / PN / N / NI
5.5 **If PN/N to 5.1, or Y/PY to 5.2 or 5.3**: Is there evidence that results were robust to the presence of missing data?		NA / Y / PY / PN / N / NI
**Risk of bias judgement**	Low	Low / Moderate / Serious / Critical / NI
Optional: What is the predicted direction of bias due to missing data?		Favours experimental / Favours comparator / Towards null /Away from null / Unpredictable

**Bias in measurement of outcomes**		
6.1 Could the outcome measure have been influenced by knowledge of the intervention received?	N	Y / PY / PN / N / NI
6.2 Were outcome assessors aware of the intervention received by study participants?	PY	Y / PY / PN / N / NI
6.3 Were the methods of outcome assessment comparable across intervention groups?	Y	Y / PY / PN / N / NI
6.4 Were any systematic errors in measurement of the outcome related to intervention received?	N	Y / PY / PN / N / NI
**Risk of bias judgement**	Low to moderate	Low / Moderate / Serious / Critical / NI
Optional: What is the predicted direction of bias due to measurement of outcomes?		Favours experimental / Favours comparator / Towards null /Away from null / Unpredictable

Bias in selection of the reported result		
Is the reported effect estimate likely to be selected, on the basis of the results, from...<br>7.1. ... multiple outcome *measurements* within the outcome domain?	n	Y / PY / PN / N / NI
7.2 ... multiple *analyses* of the intervention-outcome relationship?	PN	Y / PY / PN / N / NI
7.3 ... different *subgroups*?	PY	Y / PY / PN / N / NI
**Risk of bias judgement**	Moderate to Serious	Low / Moderate / Serious / Critical / NI
Optional: What is the predicted direction of bias due to selection of the reported result?		Favours experimental / Favours comparator / Towards null /Away from null / Unpredictable

Overall bias		
**Risk of bias judgement**	Moderate	Low / Moderate / Serious / Critical / NI
Optional: What is the overall predicted direction of bias for this outcome?		Favours experimental / Favours comparator / Towards null /Away from null / Unpredictable

Schädler, 2017

Study details

**Reference**	Schädler D, Pausch C, Heise D, et al. The effect of a novel extracorporeal cytokine hemoadsorption device on IL-6 elimination in septic patients: A randomized controlled trial. PLoS One. 2017;12(10):e0187015. Published 2017 Oct 30.

Study design

- X Individually-randomized parallel-group trial
- ☐ Cluster-randomized parallel-group trial
- ☐ Individually randomized cross-over (or other matched) trial

For the purposes of this assessment, the interventions being compared are defined as

Experimental:	6-hour CytoSorb hemoperfusion	Comparator:	No hemoperfusion

**Specify which outcome is being assessed for risk of bias**	90-day mortality (septic shock patients)
**Specify the numerical result being assessed.** In case of multiple alternative analyses being presented, specify the numeric result (e.g. RR = 1.52 (95% CI 0.83 to 2.77) and/or a reference (e.g. to a table, figure or paragraph) that uniquely defines the result being assessed.	

Is the review team's aim for this result…?

- X to assess the effect of *assignment to intervention* (the 'intention-to-treat' effect)
- ☐ to assess the effect of *adhering to intervention* (the 'per-protocol' effect)

If the aim is to assess the effect of *adhering to intervention*, select the deviations from intended intervention that should be addressed (at least one must be checked):

☐ occurrence of non-protocol interventions

☐ failures in implementing the intervention that could have affected the outcome

☐ non-adherence to their assigned intervention by trial participants

Which of the following sources were obtained to help inform the risk-of-bias assessment? (tick as many as apply)

☐ Journal article(s) with results of the trial

X Trial protocol

☐ Statistical analysis plan (SAP)

X Non-commercial trial registry record (e.g. ClinicalTrials.gov record)

☐ Company-owned trial registry record (e.g. GSK Clinical Study Register record)

☐ "Grey literature" (e.g. unpublished thesis)

☐ Conference abstract(s) about the trial

☐ Regulatory document (e.g. Clinical Study Report, Drug Approval Package)

☐ Research ethics application

☐ Grant database summary (e.g. NIH RePORTER or Research Councils UK Gateway to Research)

☐ Personal communication with trialist

☐ Personal communication with the sponsor

Risk of bias assessment

Responses underlined in green are potential markers for low risk of bias, and responses in red are potential markers for a risk of bias. Where questions relate only to sign posts to other questions, no formatting is used.

Domain 1: Risk of bias arising from the randomization process

Signalling questions	Comments	Response options
**1.1 Was the allocation sequence random?**	PN	Y / PY / PN / N / NI
**1.2 Was the allocation sequence concealed until participants were enrolled and assigned to interventions?**	PN<br>(Not concealed for 32 patients)	Y / PY / PN / N / NI
**1.3 Did baseline differences between intervention groups suggest a problem with the randomization process?**	PY	Y / PY / PN / N / NI
**Risk-of-bias judgement**	High	Low / High / Some concerns
Optional: What is the predicted direction of bias arising from the randomization process?		NA / Favours experimental / Favours comparator / Towards null /Away from null / Unpredictable

Domain 2: Risk of bias due to deviations from the intended interventions (*effect of assignment to intervention*)

Signalling questions	Comments	Response options
**2.1. Were participants aware of their assigned intervention during the trial?**	Y	Y / PY / PN / N / NI
**2.2. Were carers and people delivering the interventions aware of participants' assigned intervention during the trial?**	Y	Y / PY / PN / N / NI
**2.3. If Y/PY/NI to 2.1 or 2.2: Were there deviations from the intended intervention that arose because of the trial context?**	PN	NA / Y / PY / PN / N / NI
**2.4 If Y/PY to 2.3: Were these deviations likely to have affected the outcome?**		NA / Y / PY / PN / N / NI
**2.5. If Y/PY/NI to 2.4: Were these deviations from intended intervention balanced between groups?**		NA / Y / PY / PN / N / NI
**2.6 Was an appropriate analysis used to estimate the effect of assignment to intervention?**	Y	Y / PY / PN / N / NI
**2.7 If N/PN/NI to 2.6: Was there potential for a substantial impact (on the result) of the failure to analyse participants in the group to which they were randomized?**		NA / Y / PY / PN / N / NI
**Risk-of-bias judgement**	Low	Low / High / Some concerns

Optional: What is the predicted direction of bias due to deviations from intended interventions?		NA / Favours experimental / Favours comparator / Towards null /Away from null / Unpredictable

Domain 3: Missing outcome data

Signalling questions	Comments	Response options
**3.1 Were data for this outcome available for all, or nearly all, participants randomized?**	Py	Y / PY / PN / N / NI
**3.2 If N/PN/NI to 3.1: Is there evidence that the result was not biased by missing outcome data?**		NA / Y / PY / PN / N
**3.3 If N/PN to 3.2: Could missingness in the outcome depend on its true value?**		NA / Y / PY / PN / N / NI
**3.4 If Y/PY/NI to 3.3: Is it likely that missingness in the outcome depended on its true value?**		NA / Y / PY / PN / N / NI
**Risk-of-bias judgement**	Low	Low / High / Some concerns
Optional: What is the predicted direction of bias due to missing outcome data?		NA / Favours experimental / Favours comparator / Towards null /Away from null / Unpredictable

Domain 4: Risk of bias in measurement of the outcome

Signalling questions	Comments	Response options
**4.1 Was the method of measuring the outcome inappropriate?**	N	Y / PY / PN / N / NI
**4.2 Could measurement or ascertainment of the outcome have differed between intervention groups?**	N	Y / PY / PN / N / NI
**4.3 If N/PN/NI to 4.1 and 4.2: Were outcome assessors aware of the intervention received by study participants?**	PY	NA / Y / PY / PN / N / NI
**4.4 If Y/PY/NI to 4.3: Could assessment of the outcome have been influenced by knowledge of intervention received?**	N	NA / Y / PY / PN / N / NI
**4.5 If Y/PY/NI to 4.4: Is it likely that assessment of the outcome was influenced by knowledge of intervention received?**		NA / Y / PY / PN / N / NI
**Risk-of-bias judgement**	low	Low / High / Some concerns
Optional: What is the predicted direction of bias in measurement of the outcome?		NA / Favours experimental / Favours comparator / Towards null /Away from null / Unpredictable

Domain 5: Risk of bias in selection of the reported result

Signalling questions	Comments	Response options
**5.1 Were the data that produced this result analysed in accordance with a pre-specified analysis plan that was finalized before unblinded outcome data were available for analysis?**	NI	Y / PY / PN / N / NI
**Is the numerical result being assessed likely to have been selected, on the basis of the results, from...**		
**5.2. ... multiple eligible outcome measurements (e.g. scales, definitions, time points) within the outcome domain?**	N	Y / PY / PN / N / NI
**5.3 ... multiple eligible analyses of the data?**	PY	Y / PY / PN / N / NI
**Risk-of-bias judgement**	High	Low / High / Some concerns
Optional: What is the predicted direction of bias due to selection of the reported result?		NA / Favours experimental / Favours comparator / Towards null /Away from null / Unpredictable

Overall risk of bias

**Risk-of-bias judgement**	Some concern	Low / High / Some concerns
Optional: What is the overall predicted direction of bias for this outcome?		NA / Favours experimental / Favours comparator / Towards null /Away from null / Unpredictable

Scheer, 2017

Responses underlined in green are potential markers for low risk of bias, and responses in red are potential markers for a risk of bias. Where questions relate only to sign posts to other questions, no formatting is used.

Signalling questions	Description	Response options
**Bias due to confounding**		
1.1 Is there potential for confounding of the effect of intervention in this study?<br>**If N/PN to 1.1:** the study can be considered to be at low risk of bias due to confounding and no further signalling questions need be considered	Y	Y / PY / PN / N
**If Y/PY to 1.1**: determine whether there is a need to assess time-varying confounding:		
1.2. Was the analysis based on splitting participants' follow up time according to intervention received?<br>**If N/PN**, answer questions relating to baseline confounding (1.4 to 1.6)<br>**If Y/PY**, go to question 1.3.	N	NA / Y / PY / PN / N / NI
1.3. Were intervention discontinuations or switches likely to be related to factors that are prognostic for the outcome?<br>**If N/PN**, answer questions relating to baseline confounding (1.4 to 1.6)<br>**If Y/PY**, answer questions relating to both baseline and time-varying confounding (1.7 and 1.8)		NA / Y / PY / PN / N / NI

**Questions relating to baseline confounding only**		
1.4. Did the authors use an appropriate analysis method that controlled for all the important confounding domains?	PY	NA / Y / PY / PN / N / NI
1.5. **If Y/PY to 1.4**: Were confounding domains that were controlled for measured validly and reliably by the variables available in this study?	Y	NA / Y / PY / PN / N / NI
1.6. Did the authors control for any post-intervention variables that could have been affected by the intervention?	N	NA / Y / PY / PN / N / NI
**Questions relating to baseline and time-varying confounding**		
1.7. Did the authors use an appropriate analysis method that controlled for all the important confounding domains and for time-varying confounding?	NI	NA / Y / PY / PN / N / NI
1.8. **If Y/PY to 1.7**: Were confounding domains that were controlled for measured validly and reliably by the variables available in this study?		NA / Y / PY / PN / N / NI
**Risk of bias judgement**	Moderate	Low / Moderate / Serious / Critical / NI
Optional: What is the predicted direction of bias due to confounding?		Favours experimental / Favours comparator / Unpredictable

Bias in selection of participants into the study		
2.1. Was selection of participants into the study (or into the analysis) based on participant characteristics observed after the start of intervention?<br>**If N/PN to 2.1:** go to 2.4<br>2.2. **If Y/PY to 2.1**: Were the post-intervention variables that influenced selection likely to be associated with intervention?<br>2.3 **If Y/PY to 2.2**: Were the post-intervention variables that influenced selection likely to be influenced by the outcome or a cause of the outcome?	N	Y / PY / PN / N / NI<br><br>NA / Y / PY / PN / N / NI<br><br>NA / Y / PY / PN / N / NI
2.4. Do start of follow-up and start of intervention coincide for most participants?	Y	Y / PY / PN / N / NI
2.5. **If Y/PY to 2.2 and 2.3, or N/PN to 2.4**: Were adjustment techniques used that are likely to correct for the presence of selection biases?		NA / Y / PY / PN / N / NI
**Risk of bias judgement**	Low	Low / Moderate / Serious / Critical / NI
Optional: What is the predicted direction of bias due to selection of participants into the study?		Favours experimental / Favours comparator / Towards null /Away from null / Unpredictable

**Bias in classification of interventions**		
3.1 Were intervention groups clearly defined?	Y	Y / PY / PN / N / NI
3.2 Was the information used to define intervention groups recorded at the start of the intervention?	Y	Y / PY / PN / N / NI
3.3 Could classification of intervention status have been affected by knowledge of the outcome or risk of the outcome?	N	Y / PY / PN / N / NI
**Risk of bias judgement**	Low	Low / Moderate / Serious / Critical / NI
Optional: What is the predicted direction of bias due to classification of interventions?		Favours experimental / Favours comparator / Towards null /Away from null / Unpredictable

Bias due to deviations from intended interventions		
**If your aim for this study is to assess the effect of assignment to intervention, answer questions 4.1 and 4.2**		
4.1. Were there deviations from the intended intervention beyond what would be expected in usual practice?		Y / PY / PN / N / NI
4.2. **If Y/PY to 4.1**: Were these deviations from intended intervention unbalanced between groups *and* likely to have affected the outcome?		NA / Y / PY / PN / N / NI
**If your aim for this study is to assess the effect of starting and adhering to intervention, answer questions 4.3 to 4.6**		
4.3. Were important co-interventions balanced across intervention groups?		Y / PY / PN / N / NI
4.4. Was the intervention implemented successfully for most participants?		Y / PY / PN / N / NI
4.5. Did study participants adhere to the assigned intervention regimen?		Y / PY / PN / N / NI
4.6. **If N/PN to 4.3, 4.4 or 4.5**: Was an appropriate analysis used to estimate the effect of starting and adhering to the intervention?		NA / Y / PY / PN / N / NI
**Risk of bias judgement**		Low / Moderate / Serious / Critical / NI

Optional: What is the predicted direction of bias due to deviations from the intended interventions?		Favours experimental / Favours comparator / Towards null /Away from null / Unpredictable

Bias due to missing data		
5.1 Were outcome data available for all, or nearly all, participants?	Y	Y / PY / PN / N / NI
5.2 Were participants excluded due to missing data on intervention status?	N	Y / PY / PN / N / NI
5.3 Were participants excluded due to missing data on other variables needed for the analysis?	Y	Y / PY / PN / N / NI
5.4 **If PN/N to 5.1, or Y/PY to 5.2 or 5.3**: Are the proportion of participants and reasons for missing data similar across interventions?	PY	NA / Y / PY / PN / N / NI
5.5 **If PN/N to 5.1, or Y/PY to 5.2 or 5.3**: Is there evidence that results were robust to the presence of missing data?	NI	NA / Y / PY / PN / N / NI
**Risk of bias judgement**	Moderate	Low / Moderate / Serious / Critical / NI
Optional: What is the predicted direction of bias due to missing data?		Favours experimental / Favours comparator / Towards null /Away from null / Unpredictable

**Bias in measurement of outcomes**		
6.1 Could the outcome measure have been influenced by knowledge of the intervention received?	N	Y / PY / PN / N / NI
6.2 Were outcome assessors aware of the intervention received by study participants?	Y	Y / PY / PN / N / NI
6.3 Were the methods of outcome assessment comparable across intervention groups?	Py	Y / PY / PN / N / NI
6.4 Were any systematic errors in measurement of the outcome related to intervention received?	N	Y / PY / PN / N / NI
**Risk of bias judgement**	Low to Moderate	Low / Moderate / Serious / Critical / NI
Optional: What is the predicted direction of bias due to measurement of outcomes?		Favours experimental / Favours comparator / Towards null /Away from null / Unpredictable

Bias in selection of the reported result		
Is the reported effect estimate likely to be selected, on the basis of the results, from... 7.1. ... multiple outcome *measurements* within the outcome domain?	N	Y / PY / PN / N / NI
7.2 ... multiple *analyses* of the intervention-outcome relationship?	Py	Y / PY / PN / N / NI
7.3 ... different *subgroups*?	Pn	Y / PY / PN / N / NI
**Risk of bias judgement**	Moderate	Low / Moderate / Serious / Critical / NI
Optional: What is the predicted direction of bias due to selection of the reported result?		Favours experimental / Favours comparator / Towards null /Away from null / Unpredictable

Overall bias		
**Risk of bias judgement**	Moderate	Low / Moderate / Serious / Critical / NI
Optional: What is the overall predicted direction of bias for this outcome?		Favours experimental / Favours comparator / Towards null /Away from null / Unpredictable

Simon, 2017

Responses underlined in green are potential markers for low risk of bias, and responses in red are potential markers for a risk of bias. Where questions relate only to sign posts to other questions, no formatting is used.

Signalling questions	Description	Response options
**Bias due to confounding**		
1.1 Is there potential for confounding of the effect of intervention in this study?<br>**If N/PN to 1.1:** the study can be considered to be at low risk of bias due to confounding and no further signalling questions need be considered	Y	Y / PY / PN / N
**If Y/PY to 1.1**: determine whether there is a need to assess time-varying confounding:		
1.2. Was the analysis based on splitting participants' follow up time according to intervention received?<br>**If N/PN**, answer questions relating to baseline confounding (1.4 to 1.6)<br>**If Y/PY**, go to question 1.3.	N	NA / Y / PY / PN / N / NI
1.3. Were intervention discontinuations or switches likely to be related to factors that are prognostic for the outcome?<br>**If N/PN**, answer questions relating to baseline confounding (1.4 to 1.6)<br>**If Y/PY**, answer questions relating to both baseline and time-varying confounding (1.7 and 1.8)		NA / Y / PY / PN / N / NI

**Questions relating to baseline confounding only**		
1.4. Did the authors use an appropriate analysis method that controlled for all the important confounding domains?	Ni	NA / Y / PY / PN / N / NI
1.5. **If Y/PY to 1.4**: Were confounding domains that were controlled for measured validly and reliably by the variables available in this study?		NA / Y / PY / PN / N / NI
1.6. Did the authors control for any post-intervention variables that could have been affected by the intervention?	N	NA / Y / PY / PN / N / NI
**Questions relating to baseline and time-varying confounding**		
1.7. Did the authors use an appropriate analysis method that controlled for all the important confounding domains and for time-varying confounding?	N	NA / Y / PY / PN / N / NI
1.8. **If Y/PY to 1.7**: Were confounding domains that were controlled for measured validly and reliably by the variables available in this study?		NA / Y / PY / PN / N / NI
**Risk of bias judgement**	Serious	Low / Moderate / Serious / Critical / NI
Optional: What is the predicted direction of bias due to confounding?		Favours experimental / Favours comparator / Unpredictable

**Bias in selection of participants into the study**		
2.1. Was selection of participants into the study (or into the analysis) based on participant characteristics observed after the start of intervention?<br>**If N/PN to 2.1:** go to 2.4<br>2.2. **If Y/PY to 2.1**: Were the post-intervention variables that influenced selection likely to be associated with intervention?<br>2.3 **If Y/PY to 2.2**: Were the post-intervention variables that influenced selection likely to be influenced by the outcome or a cause of the outcome?	N	Y / PY / PN / N / NI<br><br>NA / Y / PY / PN / N / NI<br><br>NA / Y / PY / PN / N / NI
2.4. Do start of follow-up and start of intervention coincide for most participants?	Y	Y / PY / PN / N / NI
2.5. **If Y/PY to 2.2 and 2.3, or N/PN to 2.4**: Were adjustment techniques used that are likely to correct for the presence of selection biases?		NA / Y / PY / PN / N / NI
**Risk of bias judgement**	Low	Low / Moderate / Serious / Critical / NI
Optional: What is the predicted direction of bias due to selection of participants into the study?		Favours experimental / Favours comparator / Towards null /Away from null / Unpredictable

Bias in classification of interventions		
3.1 Were intervention groups clearly defined?	Y	Y / PY / PN / N / NI
3.2 Was the information used to define intervention groups recorded at the start of the intervention?	Y	Y / PY / PN / N / NI
3.3 Could classification of intervention status have been affected by knowledge of the outcome or risk of the outcome?	N	Y / PY / PN / N / NI
**Risk of bias judgement**	Low to moderate	Low / Moderate / Serious / Critical / NI
Optional: What is the predicted direction of bias due to classification of interventions?		Favours experimental / Favours comparator / Towards null /Away from null / Unpredictable

Bias due to missing data		
5.1 Were outcome data available for all, or nearly all, participants?	Y	Y / PY / PN / N / NI
5.2 Were participants excluded due to missing data on intervention status?	N	Y / PY / PN / N / NI
5.3 Were participants excluded due to missing data on other variables needed for the analysis?	PN	Y / PY / PN / N / NI
5.4 **If PN/N to 5.1, or Y/PY to 5.2 or 5.3**: Are the proportion of participants and reasons for missing data similar across interventions?		NA / Y / PY / PN / N / NI
5.5 **If PN/N to 5.1, or Y/PY to 5.2 or 5.3**: Is there evidence that results were robust to the presence of missing data?		NA / Y / PY / PN / N / NI
**Risk of bias judgement**	Low	Low / Moderate / Serious / Critical / NI
Optional: What is the predicted direction of bias due to missing data?		Favours experimental / Favours comparator / Towards null /Away from null / Unpredictable

**Bias in measurement of outcomes**		
6.1 Could the outcome measure have been influenced by knowledge of the intervention received?	N	Y / PY / PN / N / NI
6.2 Were outcome assessors aware of the intervention received by study participants?	NI	Y / PY / PN / N / NI
6.3 Were the methods of outcome assessment comparable across intervention groups?	PY	Y / PY / PN / N / NI
6.4 Were any systematic errors in measurement of the outcome related to intervention received?	N	Y / PY / PN / N / NI
**Risk of bias judgement**	Low to Moderate	Low / Moderate / Serious / Critical / NI
Optional: What is the predicted direction of bias due to measurement of outcomes?		Favours experimental / Favours comparator / Towards null /Away from null / Unpredictable

Bias in selection of the reported result		
Is the reported effect estimate likely to be selected, on the basis of the results, from...		
7.1. ... multiple outcome *measurements* within the outcome domain?	N	Y / PY / PN / N / NI
7.2 ... multiple *analyses* of the intervention-outcome relationship?	PN	Y / PY / PN / N / NI
7.3 ... different *subgroups*?	NI	Y / PY / PN / N / NI
**Risk of bias judgement**	Low to moderate	Low / Moderate / Serious / Critical / NI
Optional: What is the predicted direction of bias due to selection of the reported result?		Favours experimental / Favours comparator / Towards null /Away from null / Unpredictable

Overall bias		
**Risk of bias judgement**	Moderate	Low / Moderate / Serious / Critical / NI
Optional: What is the overall predicted direction of bias for this outcome?		Favours experimental / Favours comparator / Towards null /Away from null / Unpredictable
